# Methods for Human-Centered eHealth Development: Narrative Scoping Review

**DOI:** 10.2196/31858

**Published:** 2022-01-27

**Authors:** Hanneke Kip, Julia Keizer, Marcia C da Silva, Nienke Beerlage-de Jong, Nadine Köhle, Saskia M Kelders

**Affiliations:** 1 Centre for eHealth and Wellbeing Research Department of Psychology, Health and Technology University of Twente Enschede Netherlands; 2 Department of Research Transfore Deventer Netherlands; 3 Department of Health Technology & Services Research University of Twente Enschede Netherlands; 4 Optentia Research Focus Area North-West University Vanderbijlpark South Africa

**Keywords:** eHealth, community-based participatory research, human-centered design, CeHRes Roadmap, internet-based intervention, technological innovations

## Abstract

**Background:**

Thorough holistic development of eHealth can contribute to a good fit among the technology, its users, and the context. However, despite the availability of frameworks, not much is known about specific research activities for different aims, phases, and settings. This results in researchers having to reinvent the wheel. Consequently, there is a need to synthesize existing knowledge on research activities for participatory eHealth development processes.

**Objective:**

The 3 main goals of this review are to create an overview of the development strategies used in studies based on the CeHRes (Center for eHealth Research) Roadmap, create an overview of the goals for which these methods can be used, and provide insight into the lessons learned about these methods.

**Methods:**

We included eHealth development studies that were based on the phases and/or principles of the CeHRes Roadmap. This framework was selected because of its focus on participatory, iterative eHealth design in context and to limit the scope of this review. Data were extracted about the type of strategy used, rationale for using the strategy, research questions, and reported information on lessons learned. The most frequently mentioned lessons learned were summarized using a narrative, inductive approach.

**Results:**

In the included 160 papers, a distinction was made between overarching development *methods* (n=10) and *products* (n=7). *Methods* are used to gather new data, whereas *products* can be used to synthesize previously collected data and support the collection of new data. The identified methods were focus groups, interviews, questionnaires, usability tests, literature studies, desk research, log data analyses, card sorting, Delphi studies, and experience sampling. The identified products were prototypes, requirements, stakeholder maps, values, behavior change strategies, personas, and business models. Examples of how these methods and products were applied in the development process and information about lessons learned were provided.

**Conclusions:**

This study shows that there is a plethora of methods and products that can be used at different points in the development process and in different settings. To do justice to the complexity of eHealth development, it seems that multiple strategies should be combined. In addition, we found no evidence for an optimal single step-by-step approach to develop eHealth. Rather, researchers need to select the most suitable research methods for their research objectives, the context in which data are collected, and the characteristics of the participants. This study serves as a first step toward creating a toolkit to support researchers in applying the CeHRes Roadmap to practice. In this way, they can shape the most suitable and efficient eHealth development process.

## Introduction

### Background

Over the past years, many different types of eHealth technologies have been developed, implemented, and studied in practice. These eHealth technologies, such as web-based interventions or mobile apps, are used to support health, well-being, and health care using technology [1]. Although the *e* in eHealth illustrates the importance of technology, eHealth encompasses much more than merely adding information and communication technology (ICT). It characterizes a novel way of thinking and working, and it changes the way health care is organized [2,3]. eHealth can offer many benefits such as increased access to care, increased efficiency and quality of care, and more ownership and self-management among patients [1]. However, in practice, many of these potential benefits are not achieved. A reason for this is low uptake; many eHealth technologies are not used as often as would be expected [4]. This can be partly explained by a suboptimal fit among the characteristics of a technology, the needs and skills of the users, and the context in which the technology is used [5]. If the content of an eHealth technology does not fit with the structures of an organization and the characteristics of end users, chances of its being successfully used are low [6]. To illustrate, if a web-based intervention requires a lot of reading, it will probably not fit well within an organization that mostly treats patients with low literacy skills. This interrelationship highlights the holistic nature of eHealth, in which technology, people, and context are intertwined. For that purpose, a user-centered, iterative, and multi-method development process in which all stakeholders are actively involved is recommended [7-10]. By means of a thorough development process in which multiple research activities are combined, eHealth that provides added value for its users and context can be realized [11].

### Models for eHealth Development

There are multiple frameworks and models that can be used to guide human-centered, iterative development processes of eHealth. Well-known examples are the CeHRes (Center for eHealth Research) Roadmap [2,11]; the person-based approach [7]; the Accelerated Creation-to-Sustainment model [12], Intervention Mapping [9], the Persuasive System Design model [13], and the agile science approach [14]. Although these abstract models offer valuable guidelines and principles, they are not, and should not be, viewed as step-by-step prescriptions of eHealth development [14,15]. Rather, they should be viewed as a framework that researchers and developers use to shape their own development process and select the most appropriate research activities. However, not much is known about which research activities are most suitable for eHealth development within specific types of contexts and participants [16]. Consequently, there might be an availability bias in eHealth development: researchers might mostly use research activities that they are experienced with or those that are often described in literature [17]. However, other less-known research activities might have been a better fit with their research questions and context. To increase knowledge on how to apply development models in practice, existing eHealth frameworks could be supplemented with practical toolkits. Such toolkits could support the operationalization of the more abstract frameworks into specific research activities [15]. They can be based on experiences and lessons learned from earlier research. In this way, they could provide an overview of the kinds of development activities that can be used in the different phases of a specific eHealth development framework and offer guidelines on when and how to use these activities.

### Objective

**Figure 1:**
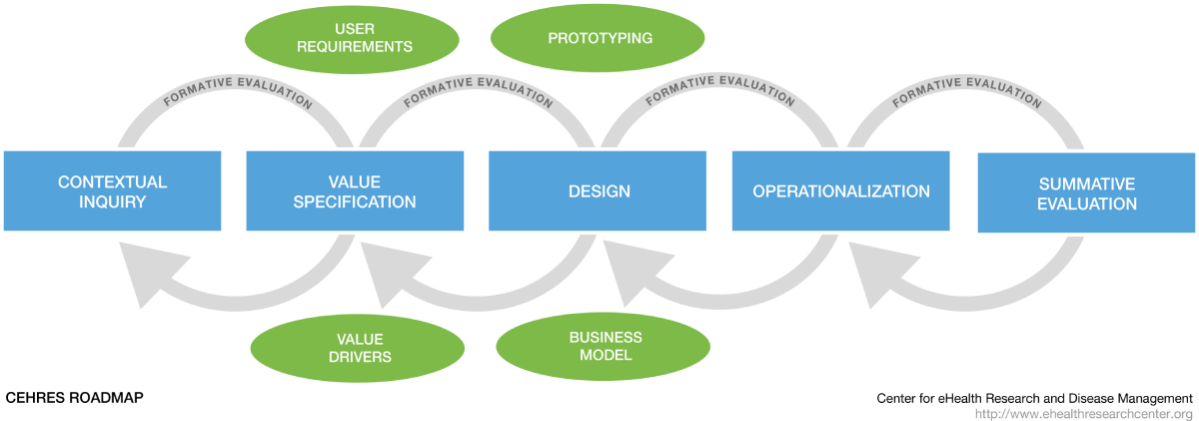
The Center for eHealth Research Roadmap [2,11,18].

In this study, we aim to create the foundation for a toolkit for a specific eHealth development framework. We provide an overview of research activities for the development of eHealth technology in context. Including studies on all eHealth development projects conducted would be a very time consuming and nearly impossible task. Therefore, this paper focuses only on studies that are based on the CeHRes Roadmap. The CeHRes Roadmap is a much-used structured framework for the development of eHealth technologies [5,16]. The results of this review can support researchers to apply the CeHRes Roadmap in practice by supporting them to select not the most obvious but the most suitable research activities. The CeHRes Roadmap (Figure 1) is based on five principles: (1) eHealth development is a participatory development process; (2) eHealth development creates new infrastructures for improving health care, health, and well-being; (3) eHealth development is intertwined with implementation; (4) eHealth development is coupled with persuasive design; and (5) eHealth development requires continuous evaluation cycles [2,11]. The CeHRes Roadmap consists of 5 intertwined phases (the contextual inquiry, value specification, design, operationalization, and summative evaluation phase) that are connected by formative evaluation cycles [11]. The first 3 phases are focused on the development of eHealth. As the CeHRes Roadmap is very comprehensive, many of these principles are also important in other (aforementioned) eHealth development models. Consequently, although this review does not cover all eHealth development models, the identified research activities and lessons learned would also be suitable for application to other eHealth development models. To provide an overview of research activities used in studies guided by the CeHRes Roadmap, this narrative scoping review focuses on the following research questions:

1. Which research activities have been used in the development process of eHealth technologies that were based on the principles of the CeHRes Roadmap?
2. With which goal(s) and in which phase(s) have these research activities been used in the development process of eHealth technologies?
3. What are the experiences with, and lessons learned from, the use of the research activities?

## Methods

### Inclusion and Exclusion Criteria

As the main goal of this study is to provide a focused overview of development activities used in the context of the CeHRes Roadmap and because no quality assessment of the to-be-included studies is required to reach this goal, a narrative scoping review was performed [19]. Records were included if they presented (part of) a development process of an eHealth technology that was based on the principles and/or phases of the CeHRes Roadmap. This refers to the use of ≥1 of the first 3 phases of the CeHRes Roadmap or explicit application of ≥1 of its principles. Consequently, studies were included if they referred to the original 2011 paper in which the CeHRes Roadmap was first introduced in either the introduction or methods section. This had to have been done in such a way that it became clear that ≥1 of the CeHRes Roadmap’s phases or principles was used to inspire the design of the study. Studies that only referred to the CeHRes Roadmap in their discussion section and/or did not contain activities for eHealth development were thus excluded. Studies that only focused on implementation and summative evaluation were excluded. Records were also excluded if they did not present any data but merely discussed abstract guidelines or models for eHealth development. Furthermore, records not written in English, Dutch, German, or Portuguese were excluded. Finally, because of the broad scope and exploratory focus of this study, only study designs from peer-reviewed journals or books were included. Student reports, preprints, and poster abstracts were excluded because they were not peer reviewed.

### Literature Search

To provide a complete overview of the studies that explicitly used or were inspired by principles of the CeHRes Roadmap, a straightforward search strategy was applied. Studies that referred to either the 2011 paper in which the CeHRes Roadmap and its principles were introduced or the new book chapter about the CeHRes Roadmap were identified in Scopus, Google Scholar, and Web of Science [2,11]. To include studies that were based on the principles of the CeHRes Roadmap but had been written before the 2011 paper, a snowball sampling strategy in which records that were coauthored by the founder of the CeHRes Roadmap (JEWC van Gemert-Pijnen) were searched in the same 3 databases. All searches were performed up until June 2021.

After removing duplicates in Covidence (Veritas Health Innovation Ltd), 3 researchers (HK, JK, and MCDS) screened the titles and abstracts using the aforementioned inclusion and exclusion criteria. As it might be possible that the development process was not fully explained in the title or abstract, the criteria were applied broadly to prevent the unjust exclusion of relevant articles. In case of doubt, a record was included to prevent overlooking relevant publications. Next, records were included for full-text screening if at least one of the authors decided to include an abstract. Full texts were assessed by 1 researcher (HK, MCDS, or JK) and, in case of doubt, discussed with one of the other researchers.

### Data Extraction and Analysis

The data extraction process was performed by 3 researchers (HK, MCDS, and JK) and based on a table developed in an earlier study, which was used to present, and reflect on, eHealth development strategies [15]. All relevant information from the included records was copied into the data extraction table. The narrative data extraction form was divided into 3 main categories with accompanying subcategories and is presented in Multimedia Appendix 1. First, information on the overall goal and type of study design of the entire paper was included. Second, information was extracted for each development activity that was reported in the record. As in participatory or human-centered eHealth development processes, nonparticipatory activities such as literature reviews can also be valuable, no distinction was made between activities in which users were and were not actively involved [11]. In other words, *nonparticipatory* activities might also be valuable or even necessary for participatory development processes. For each activity, the following information was reported in the form: research goal, target group and participants, description of research activity used, rationale for research activity, main results that were obtained by means of the activity, and phase of the CeHRes Roadmap that the research activity was used in. If the phase of the CeHRes Roadmap was not explicitly mentioned in the record, this information was deduced by the authors using the goals and methods as reported in a recent publication on the CeHRes Roadmap [11]. Third, all lessons learned about the application of the method that were reported in the records were copied into the data extraction form.

To analyze the data and answer the research questions, multiple steps were taken. To answer the first research question, an overview of research activities used in all studies was created. As activities were often named in slightly different ways, researchers formulated overarching categories for development activities by means of discussions until consensus was reached. In addition, a definition for each research activity was formulated. This definition was created by means of the information provided by the authors of the included records. If necessary, the definition was subsequently fine-tuned. This was done using other relevant literature—mainly a book that was edited by the research group of this paper’s authors [5]—and discussion among the authors of this paper. To answer the second research question, all information on the goal of a research activity, its main results, and the phase of the CeHRes Roadmap in which it was used was combined into 1 document. Researchers used this information to summarize the ways in which an activity was used and identify examples to illustrate the goals that can be achieved with the research activity. Again, if necessary, discussion among the authors took place until consensus was reached. Third, to answer the final research question on lessons learned, all extracted fragments with information about lessons learned were provided per activity; 2 researchers (HK and JK) went through these fragments separately and individually summarized the most important lessons learned. After discussion, an overview of all lessons learned was created. On the basis of this overview, the 3 most prevalent and applicable lessons learned were selected by the researchers and presented in a narrative way.

## Results

### Search Results

**Figure 2:**
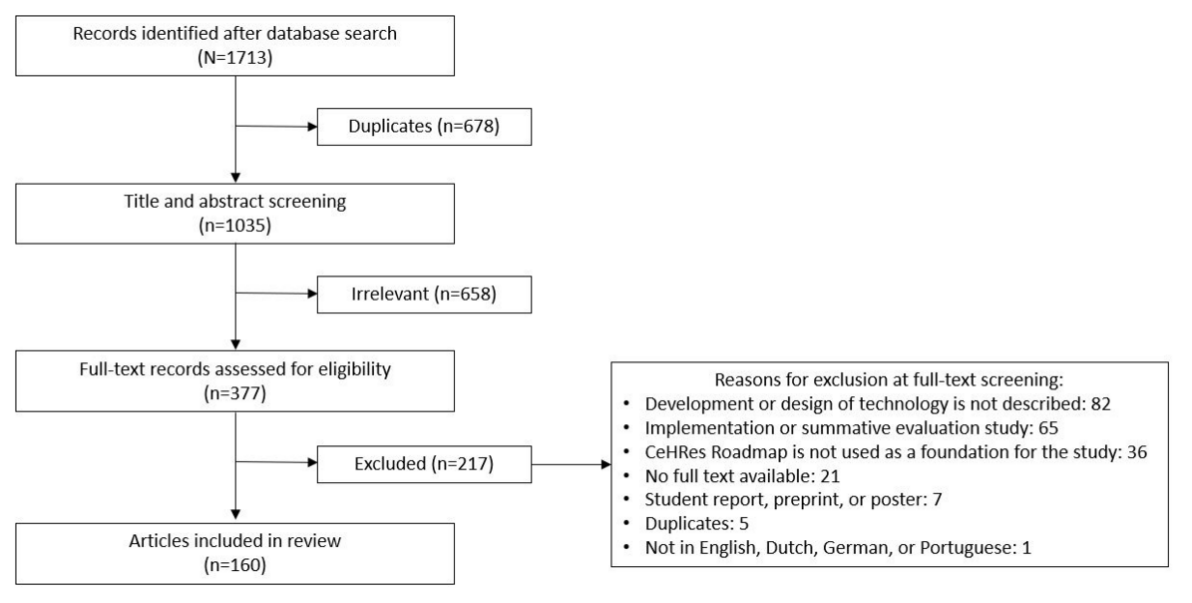
Search strategy and results. CeHRes: Center for eHealth Research.

As can be seen in Figure 2, the initial literature search yielded 1713 unique records. After title and abstract screening by 3 researchers (HK, JK, and MCDS) using the aforementioned inclusion and exclusion criteria, of the 1713 records, 377 (22.01%) remained. After full-text screening of these 377 records, 160 (42.4%) were included. The main reason for excluding full texts was a lack of specific focus on the development of an eHealth technology.

### Study Designs and Technologies

An overview was generated of the designs of all included studies (n=160). Most development studies used a multi-method approach, in which various qualitative and quantitative activities were combined (90/160, 56.3%). Other designs that were identified were qualitative cross-sectional (21/160, 13.1%), quantitative cross-sectional (18/160, 11.3%), literature studies (17/160, 10.6%), qualitative longitudinal (12/160, 7.5%), and quantitative longitudinal (2/160, 1.3%).

The included studies focused on a broad range of eHealth technologies. The main goal and described methods per study are provided in Multimedia Appendix 2 [8,15,20-176]. Most of the studies focused on web-based interventions (74/160, 46.3%) such as a web-based module for treatment of depression or a decision support tool for nurses. Mobile apps were the focus of development in 21.3% (34/160) of the studies. Apps were used, among other things, to support patients with breast cancer in doing arm and shoulder exercises and to support citizens in dealing with tick bites. In addition, virtual reality (VR) was studied in 1.9% (3/160) of the papers, which mostly focused on role-playing in treatment of patients with psychiatric disorders. Furthermore, in 16.9% (27/160) of the studies, there was no clear description of the technology, either because it was too early in the development process or because the authors did not include a description.

### Overview of Methods and Products Per Phase of the CeHRes Roadmap

During the data extraction process, a distinction had to be made between research methods and products. Methods such as interviews were used to collect new data, and products such as prototypes were based on, or summarize, previously collected data and can be used as tools to collect new data. In Table 1, an overview of all methods and products that were identified is provided, including references to the accompanying studies (Multimedia Appendix 2). In addition, for each method or product, the number of studies that used it in the contextual inquiry, value specification, or design phase is provided. As in some studies multiple methods or products were used in different phases or because methods that were relevant for >1 phase were used, the sum of the columns is not the same as the number of studies. In 30% (48/160) of the studies, the phases of the CeHRes Roadmap were mentioned explicitly; in the other studies, the CeHRes Roadmap was mostly used to inspire the design. Two authors (HK and JK) categorized the methods and products in these papers based on the definitions of the phases of the CeHRes Roadmap [11].

**Table 1:** An overview of the identified methods and products, the accompanying references, and the phases of the CeHRes (Center for eHealth Research) Roadmap in which they were categorized in the included studies (N=160).

Method or product^a^			CeHRes Roadmap phases							References
			Contextual inquiry, n (%)		Value specification, n (%)		Design, n (%)			
**Methods**										
	Focus group (n=71)	45 (63)		62 (87)		29 (41)		[8,15,20-95,177]		
	Interview (n=70)	41 (59)		32 (46)		21 (30)		[15,21,24,26,28-31,33,34,40,44,45,47,49,50,52,54-56, 58,61,67,69,70,74,76,78,79,81,83,84,88,89,91,96-129,178]		
	Questionnaire (n=51)	28 (55)		16 (31)		19 (37)		[15,24,26,29,32-34,46,49,56,60,67,69,71,76,80,84,89,96-98,101,104,107, 109,115,125-127,129-151]		
	Usability test (n=51)	0 (0)		1 (2)		67 (131)		[8,20,21,24,25,37-39,41-43,45,48-50,54,55,63,64,66,67,71,72, 75,77,80,81,84,92,98,102,104,106,107,109,110,112,113,117,118,127,131,152-156]		
	Literature study (n=43)	36 (84)		2 (5)		4 (9)		[8,20,21,27,28,38,43,46,53,55,58,69,72,75,80,81,84,92,93,99,104, 112-114,129,153,157-173]		
	Desk research (n=15)	14 (93)		3 (20)		0 (0)		[15,31,33,34,43,51,52,56,59,61,88,91,98,112,135]		
	Log data analysis (n=10)	1 (10)		0 (0)		8 (80)		[68,86,104,108,111,133,140,174-176]		
	Card sorting (n=9)	1 (11)		1 (11)		8 (89)		[8,25,34,36,70,77,98,102,125]		
	Delphi study (n=3)	1 (33)		2 (67)		0 (0)		[72,84,101]		
	Experience sampling (n=1)	1 (100)		0 (0)		0 (0)		[130]		
**Products**										
	Prototype (n=32)	0 (0)		8 (25)		29 (91)		[15,25,31,36,40,46,48,51,52,55,63,66,69,72,74,75,77,78,81,83-85, 88,92,94,96,102,107,110,113,115,121,124,127,129]		
	Requirements (n=11)	0 (0)		10 (91)		0 (0)		[20,30,31,38,39,70,91,92,114,115,129]		
	Stakeholder map (n=10)	4 (40)		4 (40)		0 (0)		[15,27,30,35,46,56,59,129,135,153]		
	Values (n=7)	0 (0)		7 (100)		0 (0)		[15,79,81,97,109,129,152]		
	Behavior change strategies (n=5)	0 (0)		1 (20)		4 (80)		[69,79,84,91,109]		
	Personas (n=5)	—^b^		1 (20)		—		[67,70,73,74,81]		
	Business model (n=4)	0 (0)		4 (100)		0 (0)		[27,62,84,99]		

^a^The sum of the times a method was used in the contextual inquiry, value specification, or design phase is higher than the number of included studies per method because in multiple studies, one method was used more than once in the development process.

^b^No relevant records were identified for the category.

### Definition, Applicability, and Lessons Learned Per Method

#### Overview

In the following sections, the definition that was generated by means of the included studies is provided for each method. In addition, different examples of how the method was used are given. Finally, the 3 most relevant lessons learned that were mentioned in the included papers are summarized.

#### Focus Group

##### Definition

Focus groups refer to meetings where qualitative data are collected by involving a relatively small number of stakeholders in a group discussion. This discussion is focused on a particular topic or set of issues, ranging from relatively unstructured *workshops* and generative design sessions to highly structured meetings.

##### Examples of Applications

Of the 160 included studies, 71 (44.4%) were focus group studies, most of which had some sort of predetermined structure. The extent to which data were systematically analyzed differed among the studies. In some, extensive coding schemes were created, whereas in others, the most important findings were summarized. Furthermore, some focus groups included a single type of stakeholder, for example, only patients, whereas others included a combination of multiple stakeholders such as therapists, patients, and technology developers. In addition, some studies included novel participants in each focus group, whereas others used recurring *coresearchers* [90]. As can be seen in Table 1, focus groups were used throughout all phases of the development process. This shows that focus groups can be used to reach a broad range of goals. Examples of these goals include the following: to identify points of improvements of the current situation, such as self-management of patients with chronic obstructive pulmonary disease (COPD) [32] or care for cerebral palsy [33]; to discuss the possibilities of a specific technology, such as the values of people with obesity regarding a to-be-developed behavior change intervention [79]; to gain insight into cognitions such as attitudes toward measures to reduce antimicrobial resistance [8]; to identify or validate values or requirements with potential end users such as health care providers [23,93]; or to collect input for the improvement of a prototype, for example, for a portal for infection control [27].

##### Lessons Learned

First, authors of multiple studies indicated that focus groups are a good way to gain more insight into the specific needs, wishes, and opinions of individuals regarding eHealth. To achieve this, focus groups can be conducted with a group of similar or very different stakeholders. Participants can bounce ideas off of each other and can directly respond to each other and can provide insight into the prevailing consensus or the range of different opinions or perspectives regarding eHealth [35-37,43,54,57,58]. However, researchers should take potential power imbalances or potentially sensitive conflicting values into account when inviting participants of a focus group. A second lesson refers to the iterative nature of eHealth development. Focus groups can be used in a *sequential* way: multiple focus groups can be conducted in a row and the goal and content of each focus group can be based on the outcomes of the previous focus groups. However, this iterative approach was said to be quite time consuming for researchers and participants [33,54,64,82,84-86,90,92]. Third, to ensure that valuable information for eHealth development is gathered, the content and form of focus groups need to be adapted, based on the topic and target group [39-41,51,55,61,87]. To illustrate, in-person focus groups are not suitable for every topic. Web-based alternatives might be considered when, for example, sensitive topics such as sexual health are discussed. Furthermore, different types of participants might require different types of focus groups. For example, focus groups with people with an intellectual disability or with older adults require a setup with more concrete examples of eHealth and might benefit from *icebreakers* and room for informal conversations [87]. In contrast, focus groups with therapists or researchers can cover more abstract topics [15].

#### Interview

##### Definition

In interviews, individuals are asked questions in a structured, semistructured, or unstructured way to obtain answers from a broad range of possible stakeholders, guided by an interview scheme.

##### Examples of Applications

Of the 160 included studies, 70 (43.8%) featured interviews that took place at multiple points in the development process. Interviews can be conducted from the start of a development process to not only analyze a problem, but also evaluate prototypes. Consequently, interviews can have a broad range of goals. Examples include identifying points of improvement for a current situation such as treatment of forensic psychiatric patients [96]; analyzing target or risk groups in, for example, tick bites [100]; identifying points of improvement for prototypes or existing (eHealth) interventions according to end users or design experts [83,84]; identifying potential barriers and facilitators for implementation later in the process, such as high costs and required skills training [78,96]; describing a current behavior and its determinants [91]; collecting experiences of participants after letting them try out an app in real life [83]; and generating or validating values and requirements [15,28,98,103].

##### Lessons Learned

First of all, in multiple papers, authors mentioned the importance of individual, in-depth interviews to incorporate the perspective of vulnerable, complex target groups such as people with dementia or severe mental illness in eHealth development [15,21,61,70,96,97,109,111,121,123,127]. This is especially important because perspectives of these underserved target groups are often overlooked in eHealth development. However, despite the benefits, including these types of target groups was found to be challenging, mostly because participating in relatively long, in-depth interviews requires a fairly high level of cognitive abilities such as attention and memory. Consequently, researchers should account for the characteristics of their target groups by, for instance, keeping the interviews as short as possible [78,128]. Another option is the use of concrete examples of eHealth technologies to account for response or recollection biases [24,52,97,121,123]. Second, although interviews can yield valuable results, a limitation is that they can offer a 1-sided picture of stakeholders’ needs and wishes regarding eHealth. Selection bias can result in a sample that is overly positive or negative [78,128]. To overcome these issues, which are related to generalizability, multiple authors recommended that interviews be combined with other methods in a multi-method or mixed methods approach [24,40,52,54,69,78,96,97,100,102,108,109,116,118,120,124,126]. This could be done by combining interviews with a small sample size with a questionnaire with a larger sample size. Although small sample sizes were not necessarily considered problematic in eHealth development, combining methods in an iterative way was suggested as a way to overcome issues with generalizability [81,127]. Third, interviews were used quite often and were generally viewed as a useful method that can be used at any point in the eHealth development process [15,21,45,54,96-98,100,105,106,109].

#### Questionnaire

##### Definition

A questionnaire can be either quantitative or qualitative; it consists of a series of open- or closed-ended questions for the purpose of gathering information from—often—a relatively large sample of respondents and can be distributed on the web or on paper.

##### Examples of Applications

Of the 160 included studies, 51 (31.9%) featured questionnaires that were applied in all phases of the development process, which means that they can be used for a broad range of goals. Examples include gathering information for stakeholder identification and analyses [135]; identifying points of improvements in conceptions and knowledge of stakeholders on infection outbreaks and antimicrobial resistance [134,178]; mapping attitudes toward technologies such as embodied conversational agents [132]; identifying needs and wishes (values) regarding a to-be-developed technology [97]; or evaluating low-fidelity prototypes, for example, scenarios on multiple possible VR interventions [15]. In questionnaires, either new questions can be generated by researchers, based on previous research, or existing questionnaires can be used, for example, the Personal Involvement Inventory, the eHealth Literacy Scale, or System Usability Scale [15,24,84,127].

##### Lessons Learned

First, in many studies, authors reflected on possible biases that might arise when using questionnaires to develop eHealth [24,69,80,97,109,115,126,134,138,139,142,146,149]. Among other things, vulnerable target groups with low literacy skills and no internet access were often hard to include in questionnaires [96]. Furthermore, some researchers used students or services such as Amazon Mechanical Turk to generate large samples; however, this raises questions about the generalizability of the results [136]. This means that results of questionnaires have to be interpreted with care and should not serve as the sole input for an eHealth technology. Second, multiple authors indicated that a questionnaire, especially one on the web, is a suitable method to quickly and efficiently collect data from different types of stakeholders and to check for differences among groups in, for example, opinions or knowledge [60,67,69,98,126,131,147,150]. A pitfall of this approach is that collecting rich in-depth information about, for example, an existing problem or a prototype is challenging. The main reason is that participants often do not provide elaborate answers to open-ended questions; in addition, it is not possible to ask probing questions [15,139,141,146]. The third lesson learned is that questionnaires need to be combined with other types of data such as interviews or focus groups to meaningfully contribute to the development process [24,26,33,60,71,96,97,126,150]. As eHealth development requires a complete picture of the current situation and needs and wishes of the stakeholders, triangulation of methods should be used. For example, products generated earlier, such as values, can be cross-referenced or interviews can be used to provide more context to the outcomes of a questionnaire.

#### Usability Testing Methods

##### Definition

Usability testing is an umbrella term that can refer to a broad range of methods such as a think-aloud method with scenarios, cognitive walkthrough, heuristic evaluation, or eye tracking. These methods are used to conduct formative evaluations of prototypes by testing them with participants such as potential users or experts. Usability refers to the extent to which a user can use a product effectively and without effort, immediately learning its use. Usability tests can be used to identify usability problems, flaws, and points of improvement or gather overall opinions.

##### Examples of Applications

Of the 160 included studies, 51 (31.9%) reported on usability tests. As these tests require a prototype that should be based on earlier research, they are often not conducted at the beginning of the development process when the scope and content of the to-be-developed eHealth technology still have to be determined. An exception is when an existing technology is evaluated in the contextual inquiry to collect input for redesign. Possible goals of usability testing are to identify points of improvements of a low- or high-fidelity prototype according to experts and/or end users (see the *Prototype* section under *Definition, Applicability, and Lessons Learned per Product*) [77,81,127,152]; to evaluate a technology’s potential to improve problems in a specific organization [77]; to analyze the way a high-fidelity prototype is used by prospective end users [24,84]; to assess whether the prototype fits the current work practice of end users such as nurses [25]; or to generate new, or further specify, values or requirements [131].

##### Lessons Learned

First, multiple authors indicated that usability tests should be conducted with a broad range of stakeholders: not only end users such as patients, but also caregivers, managers, technology developers, and experts on content and design [21,43,54,64,77,112,127,131,152,155]. Different types of participants can provide different kinds of feedback on an eHealth technology. It was suggested that experts on, for example, usability or persuasive design can be included by means of cognitive walkthroughs or heuristic evaluations, whereas users can be involved through think-aloud procedures, guided by scenarios [127]. Second, authors stated that ideally, multiple methods should be combined to paint a full picture of a prototype’s usability [24,25,64,77,80,118,152]; for example, qualitative methods such as think-aloud procedures and interviews can be combined. Qualitative approaches can also be combined with quantitative data collected by means of, for example, log data analyses, questionnaire data, or eye tracking [127]. A way to do this is by using an iterative approach based on the user-centered design framework [81,127]. Third, in multiple studies, authors indicated that values or requirements of the to-be-developed eHealth technology can be used to guide usability tests. These can be used to structure data collection by, for example, verifying whether the requirements are present in the technology, or to analyze the data by means of deductive coding using the values to ensure that everything is well aligned [8,21,42,54,113,131].

#### Literature Study

##### Definition

Although there are many ways to conduct a literature study, for example, rapid, systematic, or scoping reviews, they all aim to create an overview of a certain topic using scientific literature, often in a systematic manner.

##### Examples of Applications

Of the 160 included articles, 43 (26.9%) were literature studies, most of which were conducted at the start of a development process to create an overview or get acquainted with a specific topic. The included studies in this review ranged from relatively unstructured, quick literature scans to elaborate systematic reviews in line with PRISMA (Preferred Reporting Items for Systematic Reviews and Meta-Analyses) guidelines. An example of a literature study is a systematic review, which is often used to provide a complete, exhaustive summary of the current literature on a specific topic in a highly structured way. Another example that is often used in eHealth research is a scoping review, which offers insight into the status quo of scientific literature in a certain broad field of study by means of a systematic search of literature without paying too much attention to the quality of the studies. Possible goals of literature studies include the following: to gain insight into specific problems such as antimicrobial resistance or into broad domains such as technology in forensic psychiatry [158], to gain insight into development-related questions such as suitable persuasive features or behavior change techniques (BCTs) [159], or to develop recommendations for the design of specific interventions such as embodied conversational agents [172].

##### Lessons Learned

First, in several eHealth development studies, literature reviews were conducted quite rapidly and a systematic approach was not described. However, multiple authors indicated that it is always important to use a systematic approach when planning and executing any sort of literature review to ensure completeness of the results [158,159,166,167,173]. Second, multiple authors pointed out that often a lot of time has passed between initial data collection and publication of studies—which is especially relevant for the rapidly changing domain of eHealth. Consequently, not all state-of-the-art knowledge on technologies is published at the point of conducting the review [99,158,163,167,172]. Therefore, researchers can combine scientific literature with gray literature such as policy documents, company reports, or communication about work in progress to provide a more comprehensive overview of the current state of affairs. Third, because many literature studies in the contextual inquiry have a broad setup, it is often not possible or desirable to include a quality appraisal or only include experimental studies. If this is done, much valuable information on, for example, potential applications of a technology might be missed. In any case, it is important to reflect on the choices that were made in the reviewing process when interpreting the results [158-160].

#### Desk Research

##### Definition

Desk research refers to the nonsystematic collection of often nonscientific material such as presentations of an intervention, management reports, project documents, or activities or tasks of stakeholders. This material supports the development team in learning as much as possible about a specific topic.

##### Examples of Applications

Of the 160 included studies, 15 (9.4%) were desk research studies, which ranged from very unstructured studies, for example, talking to people or checking correspondence, to relatively structured studies, for example, a systematic analysis of the content of nonscientific documents or protocols. Desk research can be used throughout all phases of the development process when questions arise that do not require thorough, replicable research. In the included studies, desk research was used most often at the beginning of a development process to gain insight into specific fields or problems. Examples of applications include the following: to identify stakeholders, to gather information on protocols or guidelines [34,98], to create an overview of nonpublished projects on a technology in a specific sector [15], to observe existing face-to-face workshops to gain insight into their content [66], to gather correspondence for the analysis of existing communication processes [33], or to search for similar (eHealth) interventions [43].

##### Lessons Learned

First of all, authors stated that desk research should not be viewed as a synonym for randomly collecting information. Desk research should be used as a method that requires a clear research question and, if possible, a systematic search strategy that is clearly connected to the goal of the eHealth development process [15]. To illustrate, in the study by Breeman et al [81], desk research that was used to evaluate existing eHealth apps was structured by means of a newly developed evaluation tool for apps. Second, there is not necessarily a limit to the kinds of materials that can be included in desk research, depending on the research question. Some examples are apps that are available in an app store, scientific literature, protocols on cognitive behavioral therapy, written communications, policy documents, and presentations at meetings or conferences [33,98]. Third, it was suggested that desk research is a suitable method to look outside of a project’s scope to learn from the use of an eHealth technology in other domains. For example, researchers in mental health care investigated how VR is used in other settings such as hospitals or advertising [15].

#### Log Data Analysis

##### Definition

Log data are objective registrations of events that can be recorded on an individual basis, such as logging in to a website, entering a room (measured by sensors), sending a message, or performing a certain action in an app. These often large data sets can be analyzed to gain insight into behavior patterns.

##### Examples of Applications

Although log data are often used for the evaluation of the use of an already implemented eHealth technology, of the 160 included studies, 10 (6.3%) were log data studies that showed that log data can also provide valuable input for the development process. Log data can be analyzed in different ways, ranging from descriptive statistics to more complex analyses such as Markov modeling. In development, log data can, for example, be used to gain insight into use and points of improvement of (high-fidelity) prototypes [86,175] or to provide insight into dos and don’ts for the design of similar to-be-developed systems by analyzing existing similar websites [176].

##### Lessons Learned

First, authors stated that log data can be used in many ways to gain insight into online or offline behavior of prospective end users, not just eHealth use. Analyses can be performed with *real-life* log data on, for example, the use of an existing eHealth technology or with log data on the use of interaction-enabled prototypes in laboratory settings. Log data can also be collected on behavior in real-life settings that is not related to the use of an intervention but is useful for the contextual inquiry. An example is logging the number of times that doors within a nursing ward were opened [111,175]. Second, when log data on a prototype or newly developed eHealth technology are collected, researchers have to discuss with designers what data they want and how they should be collected beforehand. A well–thought out activity log protocol that describes which features should be logged should guide this debate [175]. Third, although very complicated analyses can be conducted with log data, straightforward descriptive statistics can often also be very valuable for researchers during the eHealth development process. This makes log data analysis a less complicated method than is often assumed [175].

#### Card Sorting

##### Definition

Card sorting is a method that can help design or evaluate the information architecture and structure of a technology. It can be used to structure units of information provided on cards. A distinction can be made between closed- and open-ended card sorts. In closed-ended card sorts, the main categories are provided by the researchers, whereas in open-ended card sorts, participants create their own main categories.

##### Examples of Applications

Of the 160 included studies, 9 (5.6%) involved card sorting. Card sorting is often used a bit further along the development process because it is often focused on the content and structure of an eHealth technology. Card sorting can be used to create new information structures or validate existing ones, for example, the structure of menus on websites with infection control guidelines [77,98], or to create a logical content structure that is in line with the users’ mental model, for example, for apps or web-based tools for health care workers [34].

##### Lessons Learned

Multiple authors indicated that card sorting is a fast, efficient, and cheap method to gain insight into the structure of a to-be-developed eHealth technology, especially when conducted on the web [25,70,77,98]. Web-based card sorting using programs such as Optimal Workshop software was said to be less prone to error, less labor intensive, and more efficient because larger numbers of participants can be included with less effort. Second, card sorting offers a concrete way to support people in expressing their thoughts and needs regarding an eHealth technology. This helps researchers gain more insight into how information should be structured. Third, to paint a complete picture of prospective end users’ requirements for the eHealth technology, card sorting should be combined with other methods such as interviews or focus groups [25,77,98,102]. In addition, the categories or structures that result from card sorting should be validated by using methods such as usability testing or interviews [25].

#### Delphi Study

##### Definition

A Delphi study offers a systematic way to determine consensus among various stakeholders, mostly experts. It solicits opinions from groups in an iterative process of answering questions, usually in multiple rounds.

##### Examples of Applications

Delphi studies can be used at multiple points in the eHealth development process when consensus among stakeholders—often experts—is needed. They can, for example, be used to gain insight into preferences for the eHealth technology or to reach consensus about values or requirements. Of the 160 included studies, 3 (1.9%) were Delphi studies, which used this method to identify expert recommendations for parents to reduce the risk of depression or anxiety in their children to include in the eHealth technology [72], to reach consensus on the most optimal way to integrate an eHealth technology in standard treatment [101], and to gain insight into expert opinions on relevant self-management behaviors for reducing the impact of COPD [84].

##### Lessons Learned

First of all, authors explained that Delphi studies have to consist of multiple rounds of data collection to reach consensus. This can be done using web-based questionnaires or focus groups in which participants have to participate at least two times [72]. Other types of data can be used as a starting point, such as scientific literature or outcomes from interview studies. Second, it can be challenging to recruit participants for a Delphi study because, for example, there are not many experts in a specific or new field, which can be especially relevant when studying novel applications of an eHealth technology [101]. This implies that researchers have to carefully think about whom to involve, for example, through snowball sampling, before starting with the study. Third, reaching consensus can take time and can be complex. This means that Delphi studies can require a lot of time from researchers and participants, which is not always available in eHealth development processes [101].

#### Experience Sampling

##### Definition

Experience sampling—sometimes also referred to as ecological momentary assessment (EMA)—is a structured *diary method*. It can be used to gather relevant, subjective experiences such as physical symptoms, mood, and behavior in daily life using multiple measures throughout the day using pen and paper or technology such as apps or wearables.

##### Examples of Applications

Of the 160 included studies, only 1 (0.6%) involved experience sampling, which was used to gain insight into information-seeking behavior during an EHEC (enterohemorrhagic *Escherichia coli*) outbreak [130].

##### Lessons Learned

Although experience sampling was used in only a single study, multiple lessons learned were provided. These lessons are not directly related to, but seem to be very valuable for, eHealth development. First, experience sampling was found to be a suitable method to explore an existing situation and thus to provide valuable input for further eHealth development [130]. Second, including participants who are willing and able to constantly provide input on their experience for a longer period of time can be challenging and time consuming, which also applies when it comes to ensuring that participants do not drop out during the study [130]. Third, when researchers want to study experiences during a specific event such as a virus outbreak, they ideally want to start collecting data from the beginning of the outbreak. However, in practice, this is often impossible. This can be compensated for by using a survey with retrospective questions; however, participants might be unable to correctly and completely recall their experiences [130].

### Definition, Applicability, and Lessons Learned Per Product

#### Overview

Whereas methods are used to collect new data, products are based on, or summarize, these previously collected data. They can also be used as tools to collect new data. For each identified product, the definition, ways of applying it, and a maximum of 3 lessons learned are described in the following sections.

#### Prototype

##### Definition

Prototypes are visual representations of a to-be-developed technology, ranging from low- to high-fidelity representations. Low-fidelity prototypes often do not contain much detail, allow no automatic interaction between user and prototype, and can be relatively easy to create. Examples are paper-based sketches or wireframes, possibly combined with scenarios. High-fidelity prototypes are mostly digital; often cost more time, money, and technical skills to develop; and allow for user interactions, such as programmed apps or digital, interaction-enabled prototypes.

##### Examples of Applications

Prototypes were used in 20% (32/160) of the included studies, and in these studies, most prototypes were created during the design phase. However, it is also possible to create low-fidelity prototypes early in the development process, for example, when presenting initial ideas about a technology to participants [15]. Furthermore, end users or other stakeholders can create prototypes themselves in cocreation sessions to visualize their ideas and preferences [113]. Prototypes are often based on values and requirements [15,55,113]. In addition, multiple prototypes are often created and improved based on outcomes of usability testing [52,88].

##### Lessons Learned

First, the included studies showed that there is not 1 single way to create a prototype. Methods of creating a prototype can range from very *quick and dirty* without any content to very complex, creating a highly interactive product [40,55,121]. This depends on the goal, for example, to check the overall structure of a prototype or to identify usability problems. Second, it is important to have a rough idea of the costs of developing a technology as soon as possible, mostly to prevent the final version of the prototype from seeming to be too expensive. Inclusion of technology developers from the start of the development process was recommended to prevent these problems [46,113]. Third, because major changes might be made to prototypes in an iterative development process, changes to the requirements might also be necessary to ensure that they remain in line with the prototype of the eHealth technology. This highlights the importance of an iterative approach [55,113,115]. In line with this, making major changes to prototypes could require time and resources that are not available, which might result in a suboptimal prototype [88].

#### Requirements

##### Definition

Requirements are short statements that prescribe what is *required* of a technology: “They describe what a technology should do, what data it should store or retrieve, what content it should display, and what kind of user experience it should provide” [179].

##### Examples of Applications

Requirements were formulated in 6.9% (11/160) of the included studies. They were never formulated at the beginning of the development process because they should be based on outcomes of earlier activities. The included studies showed that requirements can be based on data generated by 1 method, such as interviews, or on a combination of data from different methods and scientific literature [91]. Requirements can be based on values, where values serve as a *bridge* between the previously conducted research and the specific requirements [129]. Requirements can be used to specify previously formulated values, to serve as foundations for prototypes, or to communicate needs and wishes to developers and discuss these needs and wishes with them [15,30].

##### Lessons Learned

First of all, authors stated that requirements should be elicited in a systematic way. Ideally, multiple sources of data should be combined, such as scientific literature and qualitative data collected from multiple types of stakeholders [91,114,115]. When combining sources, development teams need to be aware of conflicting requirements, for example, a discrepancy between user needs and scientific literature [91,92]. Second, it might not be possible to include all requirements in an eHealth technology because of practical, technical, or financial limitations. However, that does not mean that it is not worth the effort to further specify them: these requirements might be incorporated in the technology at a later point in time [38,39]. Third, it was mentioned that eliciting requirements from target groups comprising patients who are vulnerable and clinically complex, such as people with psychosis or dementia, might be challenging. Consequently, researchers should carefully select methods that fit the characteristics and skills of these populations [38,39,70].

#### Stakeholder Map

##### Definition

A stakeholder map is a visualized overview of stakeholders—people or organizations who affect or are affected by an eHealth technology—and their interrelationships.

##### Examples of Applications

Stakeholder maps were reported in 6.3% (10/160) of the included studies. Stakeholders should be identified from the start of the development process, and this overview should be updated throughout the entire process. Examples of stakeholders are patients, experts on a specific topic such as depression, a commercial company that can develop apps or VR, health care providers, researchers on eHealth or the health problem at hand, and employees of other organizations that could use the intervention later [15,129,153]. The roles and tasks of these stakeholders regarding the to-be-developed eHealth technology should be identified by means of methods such as interviews or desk research.

##### Lessons Learned

First, in the included papers it was shown that stakeholder maps are not created from scratch but are based on data. Often, a stakeholder map is created by means of stakeholder identification: the systematic process of finding out who the stakeholders of an eHealth development process are. Stakeholder identification can be supplemented by a stakeholder analysis, which refers to the analysis of interdependencies, responsibilities, and stakes of the identified stakeholders. To achieve this, ideally, a combination of research methods such as questionnaires, interviews, or literature reviews is used [27,129]. Second, the studies stated that the importance of a stakeholder map should not be underestimated. Thorough investigation of stakeholders and their context is very important not only for the entire development process, but also for implementation and evaluation of the to-be-developed eHealth technology [30,35]. Consequently, the stakeholder map should be constantly updated throughout the process because new insights might arise or new stakeholders might emerge [35,59]. Third, it was considered to be important to include a wide variety of stakeholders, where researchers should look beyond their own setting. For example, when developing a VR intervention for forensic mental health care, researchers should also include stakeholders in other organizations where VR is used, such as hospitals [15,35,153].

#### Values

##### Definition

Values refer to ideals or interests of stakeholders: they specify what stakeholders want to achieve or improve by means of an eHealth technology and capture what the added value of a technology should be for the people and organization involved.

##### Examples of Applications

Of the 160 included studies, 7 (4.4%) involved values, which were created based on outcomes of previously conducted research. This means that values are often formulated later in the development process, mostly during the CeHRes Roadmap’s value specification phase, hence the name. Values remain relevant throughout the remainder of the process because they can also serve as foundations for requirements and prototypes. Values can be used to summarize or synthesize outcomes of previously conducted studies such as interviews or questionnaires. They can also serve as foundations for prototypes, implementation plans, or evaluation goals [97]. Examples of values are *improvement of skills, easy to use in current treatment, affordability, self-management*, and *positive self-image* [79,97].

##### Lessons Learned

First, authors stated that values should be formulated in such a way that they are neither too specific to prevent overlap with requirements nor too broad and vague. A shared understanding among the eHealth development team members about what values are is essential to achieve this [15,64,97,152]. Second, values should capture the whole range of stakeholder needs and wishes, not merely those of end users of the eHealth technology [81,129]. To paint a complete picture, multiple methods should be used. In addition, when new insights arise, values should be updated by a multidisciplinary research team to ensure that they continue to align with the perspectives of the key stakeholders [15,62,79,97,129]. Third, conflicting values might arise related to, for example, costs or the focus of an eHealth technology. A good way to resolve these conflicts is by discussing them with a group consisting of multiple types of stakeholders [79,97]. In addition, researchers should not view a value map as static: it might have to be adjusted based on changes in the context and users [129].

#### Behavior Change Strategies

##### Definition

Behavior change strategies such as evidence-based BCTs or persuasive elements can be integrated in the design of eHealth technologies to increase their effectiveness.

##### Examples of Applications

Of the 160 included studies, 5 (3.1%) featured behavior change strategies, which were mostly used in the design phase. The product was used to determine which theory-based methods should be included in an intervention to increase the chances of achieving behavior change. Examples are the inclusion of BCTs such as goal setting and self-monitoring in a mobile app to increase vegetable consumption [69] or the application of 3 theory-based methods and 4 accompanying strategies to influence the attitudes and skills of patients to support them in their communication with health care professionals [109]. In the study by Asbjørnsen et al [79], researchers used methods from design thinking to translate values and needs of people with obesity into persuasive features and behavior change theories such as goals and planning, personalization, and self-monitoring.

##### Lessons Learned

First, behavior change theories should ideally be combined with outcomes of human-centered design methods, instead of being mostly researcher based. However, more insight is required into how this should be done in eHealth development [79,109]. Second, operationalizing theoretical strategies into a user-friendly eHealth technology might be challenging [79,91]. To overcome this, participatory approaches such as cocreation sessions or prototyping workshops can be used to determine how theoretical working mechanisms can be translated into features of an eHealth technology [69,79]. Third, traditional behavior change theories might be too static to integrate into adaptive eHealth technologies, especially in the case of just-in-time personalized interventions. This implies that new types of theories might be required [69].

#### Persona

##### Definition

Personas are *user archetypes* that summarize a representative person from the target group. They consist of a description of different types of characteristics of a future or actual user, often in the form of a story.

##### Examples of Applications

Of the 160 included studies, 5 (3.1%) described personas. In the study by Dick et al [73], 3 personas of users of an eHealth intervention for illicit substance use were created: the heavy user, abstainer, and occasional user, whereas the study by Derks et al [70] developed personas for an intervention for people with cardiovascular disease and the study by Breeman et al [81] developed personas for an intervention for people with borderline personality disorder.

##### Lessons Learned

First of all, it is recommended to use existing guidelines and frameworks such as that of LeRouge et al [180] when developing personas for eHealth technologies. These frameworks should be combined with human-centered research methods such as focus groups [67,70,81]. Second, to structure the persona-building process, researchers need to identify characteristic categories that need to be included in the persona, in which attention should also be paid to skills and attitudes related to the to-be-developed eHealth technology. Examples are demographics and personality of the service user; their medical and psychological profile, including fears and motivations for behavior; their abilities, (technological) skills, and coping strategies; and their needs and goals [67,70,73,81]. Finally, personas were seen as a useful tool to tailor the content and design of eHealth technologies and can be connected to the requirements or to BCTs [73].

#### Business Model

##### Definition

A business model captures how an organization creates, delivers, and captures values; it describes how an organization conducts its business. It is a conceptual and analytical framework to map, discuss, and help realize the added value of an eHealth technology, as well as to determine the key factors that are associated with a sound and sustainable implementation.

##### Examples of Applications

Of the 160 included studies, 4 (3%) described business models. Often, the development of business models is initiated during the first stages of a development process because development and implementation should be intertwined [84]. However, the studies showed that a business model is not finished during development: it should be updated throughout the entire development and implementation process. An example of its application is the use of the business model canvas for an eHealth portal for infection control. This model includes the technology’s key partners, key activities and key resources, cost structure, revenue streams, value proposition, customer relationships, customer segments, and channels [27].

##### Lessons Learned

First, researchers concluded that perspectives from all important stakeholders should be accounted for in a business model. This can be done by means of focus groups with multiple stakeholders, integration of earlier collected data, or in-depth interviews [84]. When collecting input for the business model, it was indicated that questions to participants should be very concrete because abstract questions will yield equally abstract and thus less useful answers [99]. Second, experience has shown that creating a business model is very time consuming, partly because there are no business models specifically for eHealth yet [27,99]. Third, a business model often does not have a fixed end and needs to be adapted continuously throughout the implementation and evaluation phases of an eHealth technology [27].

## Discussion

### Principal Findings

The main goal of this narrative scoping review is to create an initial overview of the methods used in eHealth development processes guided by the CeHRes Roadmap. Furthermore, we aim to identify for what purposes and in which phases these methods are used and provide an overview of the most relevant lessons learned. During the analysis, it became clear that a distinction between development methods and products can be made. In the 160 included studies, 10 overarching methods and 7 products were identified. Most of the identified methods were used in all 3 development phases of the CeHRes Roadmap. They were used for a broad range of goals, underlining the many different possibilities that exist for eHealth development. The lessons learned showed that most authors agreed that the methods and products contributed to eHealth development in a positive way by providing more insight into the users and context. However, a critical reflection on the methods or products and accompanying conclusions related to a method or product not being suitable were often not provided in the included studies. Regardless, authors mentioned multiple barriers and limitations that they had to account for, which differ per method and product. On the basis of the many lessons learned that we identified, there seems to be ample experience with, and knowledge about, different types of development methods. However, this knowledge remains mostly segregated and there could be more room for critical reflection on the suitability of a research activity. Furthermore, several potentially useful methods and products such as experience sampling, personas, or behavior change strategies seem to be underrepresented in the included studies. This underlines the need for better integration and broader dissemination of knowledge on eHealth development. Integrating and sharing knowledge can enable researchers to select the most fitting method or product, as opposed to using the one that is most easily available or well-known.

### Comparison With Prior Work

#### Methods Versus Products

In this review, a distinction was made between methods and products in eHealth development. This distinction does justice to the diverging and converging nature of eHealth development. This is in line with design-thinking approaches such as the double diamond model, which pays attention to diverging, for example, by means of collecting data, and converging, for example, by means of integrating the findings in a set of requirements or a prototype [15,181]. Similarly, based on our findings, we recommend that methods and products should be seen not as separate activities but as 2 sides of the same eHealth development coin. This review showed that methods such as interviews, questionnaires, or literature studies are used to collect new data. These data can be translated into concrete products to synthesize collected data and to support and facilitate subsequent collection of new data. Thus, these products can serve as stepping stones among data collection methods. An example of using products as a synthesis approach is the formulation of values based on previously conducted interviews and questionnaires [15,97]. An example of the use of products to collect novel data is the use of a low-fidelity prototype in usability testing to gain insights into prospective users’ needs and wishes [131]. The combination of methods and products illustrates how researchers can continuously check assumptions and decisions with stakeholders in a concrete, specific way by means of formative evaluation cycles [11]. However, currently, the terminology used in literature does not reflect the distinction among the different types of development activities. Every development-related activity is referred to as a method, or products are only briefly described or mentioned as a tool within a method. On the basis of this review, we suggest that researchers should make an explicit distinction between methods and products when reporting on their eHealth development process.

#### Iterative eHealth Development

This review showed that there is no single ideal way to conduct a development process. The included studies illustrate that there are multiple methods that can be used in many different ways, for many different goals, and at many different points in the development process. To illustrate, for identifying points of improvement in a current situation according to stakeholders, researchers might use interviews, focus groups, and questionnaires. In addition, values and requirements can be elicited by means of both an interview study and focus groups in which prototypes are evaluated [96,97,178,179]. Consequently, multiple methods can be used to reach similar goals. This implies that creating an ideal, step-by-step guideline with predetermined methods is neither possible nor desirable because this would suggest that there is a single most optimal way to conduct a development process. However, this conclusion does not mean that *anything goes*. On the basis of the lessons learned, it can be concluded that a specific method or product should fit the context in which the data collection takes place as well as the characteristics and skills of the participants and the outcomes of earlier development activities [182]. To illustrate, although in-depth interviews might be a useful way to gather information from health care workers and experts, they seem to be less suitable for people with dementia because of the cognitive skills that would be required [48,61,178]. In addition, although these approaches can be used to collect valuable data, they also illustrate that over the past 10 years, there has not been much innovation in the methods and products used in eHealth development. This is especially striking considering the rapid changes in the possibilities of eHealth technologies such as mobile apps or VR over the past years. Scientific methods such as literature reviews, focus groups, and interviews have been used very often, whereas innovative methods of a more participatory nature, for example, generative methods such as photo diaries and mood boards, are hardly used, despite their potential added value.

In addition, this review has shown that often, multiple methods can—and should—be combined to paint a full picture of the current situation and possibilities of eHealth. If done well, the use of multiple methods to incorporate the stakeholders’ perspective throughout the entire process will result in an eHealth technology that fits their needs, wishes, skills, and context. This fit among technology, stakeholders, and context will also increase the chances of the uptake of eHealth in practice. Methods can be used in different ways to include the stakeholder perspective. On the one hand, methods can be used to validate findings; for example, interviews can be used to provide more context to the results of a literature review [15]. On the other hand, methods and products can also be used in a complementary way; for example, although usability tests can be used to elicit requirements from the end user, they do not provide insight into the demands of the organization. This knowledge can be obtained by means of complementary focus groups with managers. Consequently, when selecting methods throughout the process, researchers should account for the suitability of the methods and products for their specific context, participants, and earlier findings of the development process. Furthermore, this review showed that not all methods used in human-centered development processes have to be participatory to be useful; for example, literature reviews or log data analyses can also be valuable. Nonparticipatory methods can be complemented with human-centered methods such as interviews or focus groups to form a coherent whole.

Finally, the lessons learned illustrated that when shaping a multi-method or mixed methods development process, an iterative and flexible development approach is key [33,54,55,64,113,115]. Ideally, decisions for the next development activities should be based on findings, new insights, and experiences that arose from previous activities. Such an iterative approach ensures that the development process fits the context and the people involved. This is in line with *agile science*, which states that eHealth development should be iterative and flexible, with short sprints to allow for constant changes to the process and products [14,183]. An agile approach requires a flexible mindset of researchers because it is often not possible to plan the entire development process from the start. To support researchers, the results of this review can serve as input for the creation of a *CeHRes Roadmap Toolkit* for selecting the most appropriate and fitting methods, as opposed to choosing the method that they are most familiar with. To broaden the scope of this toolkit, it could be complemented with other research, for example, reviews such as this one, but ones that are focused on other frameworks. Another way to broaden the toolkit is by asking experts on, for example, human-centered design and eHealth development about other potentially useful methods. Such a broader toolkit can support researchers in adapting their development process in case of contextual changes or unexpected or new findings [14,183]. This can facilitate the iterative nature of the development process.

#### Points of Improvement for eHealth Development Models

On the basis of the findings of this review, multiple points of improvement for the CeHRes Roadmap can be formulated. These can also be partly applied to other eHealth development models. First, it is important to note that most studies did not use all phases of the CeHRes Roadmap; rather, they *cherry-picked* the most relevant phases or principles to use in their work. Often, the CeHRes Roadmap was combined with other development approaches such as design thinking or the Behavior Change Wheel. This illustrates that in eHealth development, (parts of) multiple models, theories, and approaches can be used to complement each other [91]. Second, although business modeling and value specification are important elements of the CeHRes Roadmap, they were underrepresented in the included studies. This indicates that in general, researchers might require more guidelines on how to not only develop an intervention, but also think about its implementation in practice from the start of development. Third, a main point of improvement for eHealth development using the CeHRes Roadmap is related to the use of behavior change theory: this approach was underrepresented in the included studies [81]. This is quite surprising, especially considering the relationship between behavior change theory and effectiveness of (eHealth) interventions [184,185]. A possible explanation for this gap is that design-oriented, development models such as the CeHRes Roadmap do not explicitly *force* or nudge developers to incorporate theory, as opposed to more *human-centered* activities such as stakeholder identification or prototyping. Consequently, separate goals or activities might be added to the CeHRes Roadmap to support developers in incorporating behavior change theory [186,187]. In line with this, there is a need for more guidelines on how behavior change theory can be combined or complemented with persuasive design features. In addition, there is a need for more research on how this can in turn be connected to stakeholders’ values and requirements [79,81,159]. An important point of attention in regard to this is that there is still much debate about the suitability of existing behavior change models for eHealth. Are existing theories suitable, or are they too static for highly personalized eHealth technologies? In other words: is there a need for more dynamic, personalized models for behavior change [188]? A final recommendation is related to applying development models to practice. As conducting development processes in practice is often very complex and challenging, multiple authors recommended forming a multidisciplinary development team. Such a team can consist of, for example, researchers, patients, health care providers, technology developers, designers, and managers [15,88,90]. A multidisciplinary team can prevent a top-down approach and tunnel vision; ensure that a constant eye is kept on the context; facilitate the coordination of large, complex development processes by combining skills and knowledge; and even support implementation of the to-be-developed eHealth technology in practice [182].

### Strengths and Limitations

The main goal of this explorative narrative scoping review is to create an overview of development methods used in eHealth development guided by the CeHRes Roadmap. Although the CeHRes Roadmap is viewed as a suitable framework to guide eHealth development, there are many more useful development models. Because of our focus on studies that used the CeHRes Roadmap, several other suitable research methods or products might have been overlooked. Examples of such methods are observations, EMAs, or eye tracking [189-191]. Therefore, this overview of development methods and products is neither exhaustive nor unconditionally generalizable. Regardless, because of the interdisciplinary and broad applicability of the CeHRes Roadmap, much of the knowledge generated in this review is also relevant for other development models. However, creating a broader eHealth development toolkit would require additional similar reviews in which other development frameworks are put central. Subsequent analysis of differences and similarities among these reviews might also allow for generalization of knowledge. In line with this, because providing an elaborate, in-depth reflection on the included methods and products is beyond the scope of this paper, the provided lessons learned in this narrative scoping review are also not exhaustive. To do justice to the possibilities of all mentioned methods and products, a separate review per method is required. In addition, because of the goal and broad nature of our scoping review, we did not assess the quality of the included studies. The main reason for this was that the quality of a study does not say anything about the quality of a development method or product. In other words, if a study in which interviews were used in a contextual inquiry is of extremely high methodological quality, this does not automatically mean that interviews in themselves are always the most suitable method for contextual inquiries. On the basis of the reported lessons learned, researchers have to make their own choice for the most suitable method or product for their process. Furthermore, a pitfall of the qualitative approach that was used to describe the methods and products is that it is prone to potential bias of the researchers. To overcome this, the screening and summarizing processes were set up in a systematic way and conducted by 3 researchers. In addition, all findings were discussed among, and critically reflected upon by, the coauthors—all with ample experience in multidisciplinary, human-centered eHealth development. Finally, as is the case in any review, not all relevant information on development methods might have been published. On the one hand, this might be because, too often, development studies are not viewed as full-fledged research and are simply not published. An example of this is a stakeholder analysis, which is often conducted but hardly ever included in publications [135]. On the other hand, we noticed that there is much difference between the clarity and extensiveness of the way authors reported and reflected on development activities. Suboptimal or incomplete reflection by authors resulted in some lessons learned that remained quite general and were not specifically focused on eHealth development. In general, this might have influenced our findings and highlights the need for a larger number of, and more comprehensive, papers on eHealth development.

### Future Directions

As stated earlier, the results of this review should be viewed not as an exhaustive overview of all existing eHealth development methods and products but as an initial overview of activities related to research based on the CeHRes Roadmap. First, in multiple studies, the methods used were described in very general terms or the papers only described a single method from a larger development process [78]. Consequently, additional interviews, focus groups, or questionnaires—the choice of method naturally depending on the type of context and characteristics of the users—can be conducted with researchers to gain more in-depth insight into their experiences with eHealth development. This can also be extended to experts working in practice because innovation in human-centered design often is initiated in practice. In line with this, more specific reviews or viewpoint papers on single development methods or products might be conducted.

Second, a more general recommendation is related to the method of reporting. On the basis of the included studies, it becomes clear that there are many differences in the way authors report on development processes. In addition, as can be seen in the relatively low number of reported products compared with reported methods, there is room for improvement in the description of products used. We recommend that future development studies explicitly substantiate choices for, and critically reflect on, the methods and products used. Future research might explore the desirability and feasibility of a standardized guideline for development studies in line with the standards for reporting on, for example, randomized controlled trials (CONSORT [Consolidated Standards of Reporting Trials]) [192], reviews (PRISMA) [193], or qualitative research such as interviews (Consolidated Criteria for Reporting Qualitative Studies) [194]. On the basis of our findings, we recommend that researchers at least report on (1) the specific goal of the method, (2) a replicable description of the materials and procedure of data collection, (3) the rationale behind choosing the method, (4) the participants, (5) the setting, (6) the main results and conclusions, and (7) the most important lessons learned about the use of the method within the specific context [24]. In case of products, we recommend that they be described in a clear way and, if possible, that the products themselves be provided in the paper or appendix. Examples are screenshots of prototypes, a list with values, and an overview of stakeholders. In addition, multiple included studies showed that a visualization of the development process might also be of added value [15].

Third, the COVID-19 pandemic has shown us the potential of *online* research. Organizing web-based usability testing, focus groups, or interviews is sometimes not only necessary, but they can also have practical and even substantive advantages [87]. Future research can focus on if and how *research from a distance* can be applied to make eHealth development more efficient and effective. Finally, the results from this review can be used in the development of a digital *CeHRes Roadmap Toolkit*. This can be achieved by means of a human-centered development process in which end users—in this case, researchers and developers—are actively involved using suitable methods. Before a more comprehensive and exhaustive eHealth toolkit can be developed, there is a need for additional reviews that take a similar approach as this review but focus on other development models such as intervention mapping or user-centered design. This might result in a more exhaustive overview of other development methods and products that were not represented in this review. Furthermore, in line with the interdisciplinary nature of eHealth development, this eHealth toolkit can also be based on existing, more generic toolkits such as the Communication and Media Design methods pack [195] and the Human-Centered Design Toolkit [196]. Developing a broad eHealth toolkit requires an interdisciplinary approach in which designers with both a research and commercial background, psychologists, health scientists, other relevant stakeholders, and researchers combine their expertise. Including expertise from commercial design companies might be useful or even necessary to innovate eHealth development and go beyond the more traditional, scientific approaches.

### Conclusions

This narrative scoping review has resulted in an initial overview of multiple methods and products that can be used for development of eHealth, guided by the CeHRes Roadmap. It can be concluded that there is not a single straightforward, step-by-step way to conduct a development process: the way a process is shaped depends on the type of technology, context, and stakeholders. To support researchers in selecting the most appropriate and fitting methods and products for their development process, it is recommended to develop an eHealth development toolkit. To achieve this ultimate aim, there is a need not only for more research, but also for close collaboration with industry. In this way, eHealth development becomes less reliant on traditional research approaches such as literature reviews or interviews. Researchers can be challenged to create more creative and innovative development processes by using methods and products that are developed by industry. This review can serve as a blueprint for further reviews in which different frameworks are put central. In general, the decision for the method or product should be based on three different elements: (1) the specific goal of the current development phase, (2) the characteristics of the participants, and (3) practical requirements from the context. In this way, eHealth development processes can become more flexible and will be professionalized further. This in turn might result in more effective, personalized eHealth technologies that fit with, and are of added value for, the users and their context.

## Appendix

Multimedia Appendix 1 Data extraction form.

Multimedia Appendix 2 Main goal of the study and described methods and products per included record.

## Abbreviations

BCT: behavior change technique
CeHRes: Center for eHealth Research
CONSORT: Consolidated Standards of Reporting Trials
COPD: chronic obstructive pulmonary disease
EMA: ecological momentary assessment
EHEC: enterohemorrhagic Escherichia coli
ICT: information and communication technology
PRISMA: Preferred Reporting Items for Systematic Reviews and Meta-Analyses
VR: virtual reality
