# Supplementary material for: Methods for Human-Centered eHealth Development: Narrative Scoping Review
Source: J Med Internet Res. 2022 Jan 27;24(1):e31858. doi: 10.2196/31858 (PMC8832261; doi:10.2196/31858)
Supplement: Multimedia Appendix 1 [file jmir_v24i1e31858_app1.docx]

**Multimedia Appendix 1 – Data extraction form**

| **Study: author and title** | **Include?** | **Reason for not including/doubt** |
| --- | --- | --- |
| … | Yes/no/maybe | … |

| **Research question/goal of entire study** | **Study Design** | **Explanation study design** | **Technology studied** | **Roadmap explicitly mentioned?** |
| --- | --- | --- | --- | --- |
| … | Qualitative cross-sectional/ Qualitative longitudinal/ Quantitative cross-sectional/ Quantitative longitudinal/  Mixed or multiple methods/  Literature study | *Optional* | … | Yes/no |

| **Research goal of method** | **Target group(s)/**  **participants** | **Method used** | **Rationale for method** | **Main results (from abstract)** | **Phase of Roadmap** | **Lessons** |
| --- | --- | --- | --- | --- | --- | --- |
| … | … | … | … | … | Contextual inquiry/  Value specification/  Design | … |
|  |  |  |  |  |  | … |
|  |  |  |  |  |  | … |
