# Supplementary material for: Methods for Human-Centered eHealth Development: Narrative Scoping Review
Source: J Med Internet Res. 2022 Jan 27;24(1):e31858. doi: 10.2196/31858 (PMC8832261; doi:10.2196/31858)
Supplement: Multimedia Appendix 2 [file jmir_v24i1e31858_app2.pdf]

Multimedia Appendix 2 – Main goal of the study and described methods and products per included record

Authors	Year	Reference	Goal of the study	Included methods and products
Al-Itejawi et al.	2016	[1]	To develop a patient decision aid and to prepare an overview of requirements for implementation.	Focus groups, interviews, usability tests
Amann et al.	2020	[2]	Taking spinal cord injury as a case in point, the overall objectives of this study were to identify the perceived benefits of a co-designed self-management app that could promote its uptake and to explore the factors that may impede adoption.	Interviews, focus group
Ammerlaan et al.	2016	[3]	To provide a systematic and comprehensive description of the theoretical considerations for building a Web-based, expert, patient-guided, and tailored intervention for adult patients with a rheumatic disease. Also, to present the results of a usability study on the feasibility of this intervention, and its study design in order to measure the effectiveness.	Questionnaire, focus groups
Asbjørnsen et al.	2019	[4]	This review aimed to identify BCTs and PSD principles applied in eHealth interventions to support weight loss and weight loss maintenance, as well as techniques and principles applied to stimulate motivation and adherence for long-term weight loss maintenance.	Literature study
Asbjørnsen et al.	2020	[5]	The study sought to identify the values and needs of people with obesity aiming to maintain weight after weight loss, and to identify PSD principles, BCTs, and design requirements that potentially enable an eHealth intervention to meet end user values and needs.	Interviews, focus groups, behavior change strategies, values
Askedal et al.	2017	[6]	This study used a qualitative approach to identify and investigate contradictory stakeholder interests in the early phase of a municipal eHealth project.	Desk research, stakeholder map, focus groups
Backere, de et al.	2014	[7]	To present the methodologies to develop a cloud-based semantic system, offering valuable information and knowledge-based services.	Desk research, interviews, personas, focus groups, prototype
Beaujean et al.	2013	[8]	We aimed to identify the group of individuals within the general Dutch population that are at high risk of being bitten by a tick or developing LD and to describe their characteristics, knowledge, and perceptions.	Interviews
Beerlage-de Jong et al.	2014	[9]	Offering a (practical) method for the integration of two eHealth development frameworks (User-Centered Design and Persuasive System Design model)	Focus group, questionnaire, interviews, usability tests
Beerlage-de Jong et al.	2017	[10]	Our aim was to demonstrate why and how participatory development (involving end-users and other stakeholders) can contribute to the success of clinical decision support systems (CDSS) in antimicrobial stewardship.	Usability tests, prototype, questionnaire,
Beerlage-de Jong et al.	2017	[11]	To describe the Roadmap activities that we executed in the eZoon-project in the different phases of the project, and which knowledge they yielded.	Stakeholder map, interviews, questionnaire, card sorting,
Beerlage-de Jong, et al.	2017	[12]	In this position paper we describe i) how a socio-technical multidisciplinary approach (based on the CeHRes Roadmap) can be applied in the development and implementation of Antimicrobial Stewardship technologies and ii) how this approach can be of value to support Antimicrobial Stewardship in practice. AND In this position paper	Literature study, prototype, usability tests, focus

			we describe the lessons learned from research and practice to guide future developments of technology based ASP interventions.	groups, card sorting, stakeholder map
Belt, van de et al.	2013	[13]	To determine the preferences of the general population in the Netherlands regarding the use of the Internet and social media in health care.	Questionnaire
Bernecker et al.	2017	[14]	The aim of our study was to determine the level of public interest in a new mental health intervention that incorporates elements of self-help and peer counseling and that is disseminated via a Web-based training course; to identify predictors of interest in the program; and to identify consumer preferences for features of Web-based courses and peer support programs.	Questionnaire
Boele et al.	2017	[15]	We aimed to explore PBT caregivers' satisfaction with the current supportive care provision, as well as their thoughts on monitoring their care issues with both paper-based and digital instruments.	Interviews
Bogza et al.	2020	[16]	This study aimed to incorporate features that best support values clarification and adjust the level of detail of a web-based decision aid for individuals with mild cognitive impairment.	Literature study, prototype, usability tests, questionnaire, focus group
Børøsund et al.	2018	[17]	To develop an evidence-based stress management intervention for patients living with cancer that can be delivered electronically with wide reach and dissemination.	Interviews, focus groups, usability tests, prototypes
Bossen et al.	2016	[18]	The first aim was to develop a blended exercise therapy intervention for patients with knee and hip osteoarthritis that matches the values of the users and that can be implemented in the daily routine of physical therapists. The second aim was to investigate the feasibility through interviews and a pilot study.	Focus groups, interviews
Breeman et al.	2021	[19]	The goals of this study were threefold: to uncover stakeholders' needs and preferences, to translate these to core values, and develop eHealth technology based on these core values. Our primary research question is: What type of eHealth application to support healthy living among people with (a high risk of) CVD would provide the greatest benefit for all stakeholders?	Literature study, interviews, usability tests, focus groups, desk research, values, personas, prototypes,
Bruinessen, van et al.	2014	[20]	The objective of this project was to apply a bottom-up inspired procedure to develop a patient-centered intervention with corresponding evaluation and implementation plan.	Questionnaires, interviews, behavior change strategies, usability tests
Carr et al.	2017	[21]	To identify the needs and requirements of the end users, to inform the development of a user-interface to translate an existing evidence-based decision support tool into a practical and usable interface for health service planning for osteoarthritis (OA) care.	Focus groups
Cheung et al.	2018	[22]	To illustrate a mixed-method usability evaluation of an economic decision-support tool for tobacco control, using the EQUIPT ROI tool prototype as a case study.	Usability tests, questionnaire
Cnossen et al.	2015	[23]	To develop a Web-based self-care program for patients after total laryngectomy according to a participatory design approach.	Focus group, prototype, usability test
Corbett et al.	2017	[24]	What characteristics of business model design in precare contribute to the convenience and quality of eHealth services provided by a health professional to a client?	Literature study, desk research, focus groups, usability tests

Crane et al.	2017	[25]	The aim of this study is to explore user views toward an app to help people reduce their consumption of alcohol and determine whether the BCTs are acceptable and feasible to users and how they might be improved	Usability tests, interviews
Cruz-Martínez et al.	2018	[26]	To deliver recommendations to further improve the system in preparation for a large scale implementation.	Questionnaire, interviews, usability tests, log data analysis, literature study
Da Costa et al.	2017	[27]	To guide the development of Healthydads.ca, a website designed to enhance mental health and healthy behaviors in expectant fathers, a needs assessment was conducted to identify fathers' perspectives of barriers to seeking help for emotional wellness, informational needs, and factors affecting the decision to visit such a website.	Questionnaire
D'adderoi et al.	2020	[28]	The aim of this study was to investigate the features that are judged to be important for engagement with a physical activity mobile app and the reasons for their importance.	Focus groups
Derks et al.	2017	[29]	Using a modified UXD-framework, we investigated the need amongst BPD-patients and therapists for an ambulatory biofeedback e-coaching app that supports patients in learning to better recognise changes in their arousal level.	Interviews, card sorting, persona
Derks et al.	2019	[30]	The aim of the study was to develop an ambulatory biofeedback app for mental health care that helps with learning to better recognize changes in personal emotional arousal and increases emotional awareness.	Usability tests, questionnaire, interviews, prototypes
Dick et al.	2020	[31]	This paper describes the process and contributions of a persona-building approach in the development of a digital behaviour change intervention tailored to the needs of third-level students.	Focus groups, personas
Dijk, van de et al.	2013	[32]	To explore the requirements of older people for eHealth support, and through this to promote self-management and enhance quality of life.	Focus groups, questionnaires
Doty et al.	2020	[33]	Guided by the CeHRes Roadmap, our objective was to apply a community-based participatory research (CBPR) approach to mobile app development. We present a formative evaluation to inform the design of an eHealth mobile app for Latinx parents of adolescents based on a face-to-face parenting program, Padres Informados/Jovenes Preparados (PIJP).	Questionnaire, interviews, requirements, prototype
Downing et al.	2016	[34]	This study investigated views of mothers from disadvantaged urban and regional areas (i.e., beyond major capital cities) as potential end users of child active play and screen time behaviour change interventions, with a focus on text messaging and web-based delivery platforms.	Interviews
Duman-Lubberding et al.	2015	[35]	To investigate health care professionals' perspectives toward follow-up care and an eHealth application, OncoKompas, in follow-up cancer care that monitors quality of life via PROs, followed by automatically generated tailored feedback and personalized advice on supportive care.	Interviews, prototypes, usability tests
Duplaga	2016	[36]	To assess nurses' opinions and to analyze the predictors of their acceptance of ehealth features relevant to patient empowerment with a strong focus on chronic care.	Questionnaire
Dyk, van et al.	2012	[37]	The purpose of this paper is to analyse the commonalities and differences of two models (CeHRes Roadmap and Telemedicine Maturity Model) , and to explore how they can be used to complement each other.	Literature study, focus groups, requirements, prototypes

Ehrler et al.	2019	[38]	To present the design, development, and testing of a smartphone app for nurses guided by an adapted software development life cycle model that takes into consideration the complexity and constraints of a health care setting.	Focus group, card sorting, prototypes, usability tests
Fico et al.	2015	[39]	The work presented in this paper describe the methodologies used in the context of two multidisciplinary research projects, METABO and MOSAIC.	Focus groups, business model
Fico et al.	2015	[40]	The work presented in this paper describe the methodologies used in the context of a multidisciplinary research project and provides an overview of the preliminary results.	Focus groups, usability tests, prototype
Fico et al.	2019	[41]	To develop new computer models, and implement them in tools to support the detection and prediction of T2D onset and related complications, in different healthcare settings (e.g. hospitals, clinical centres and health agencies).	Focus groups, prototypes, questionnaire
Fledderus et al.	2015	[42]	In this study an online, automated relapse-prevention program based on acceptance and commitment therapy (ACT)—both as a website and as a mobile app—was developed and evaluated. At each step of the development, end users (ie, chronic pain patients) were consulted in order to fully address their needs.	Focus groups, interviews, usability tests, prototype
Floch et al.	2018	[43]	To develop and evaluate an app ecosystem for self-management in systic fibrosis.	Interviews, prototypes
Galpin et al.	2017	[44]	The first objective was to examine the utility of an online user design group for generating insight for the creation of Web-based health resources. We sought to explore the advantages and limitations of this approach. The second objective was to analyze what women want from a Web-based resource for breast cancer screening.	Focus groups, questionnaires
Gemert-Pijnen, van et al.	2016	[45]	In this chapter we elaborate on the use of the CeHRes roadmap for dementia care. First, we will describe the CeHRes roadmap and its principles for development, then we present a case study to demonstrate how the roadmap was used in practice, and finally we will reflect upon the case study by means of lessons learned.	Focus groups, literature study, interviews, usability tests
Gemert-Pijnen, van et al.	2011	[46]	To investigate the use of a web-based triage system in primary care, and compliance with the medical advice delivered by it.	Interviews, card sorting, prototypes, usability tests
Gemert-Pijnen, van et al.	2014	[47]	The aims of this study were (1) to illustrate how log data can be used to understand the uptake of the content of a Web-based intervention that is based on the acceptance and commitment therapy (ACT) and (2) to discover how log data can be of value for improving the incorporation of content in Web-based interventions	Log data analysis
Geraghty et al.	2016	[48]	To develop an unguided e-mental health intervention for distress in primary care patients, drawing on meta-theory of psychotherapeutic change and utilizing the person-based approach (PBA) to guide iterative qualitative piloting with patients.	Interviews, prototypes, usability tests
Gilbert et al.	2016	[49]	This paper describes the planning, development, and usability testing of a novel Web-based testing service, GetCheckedOnline (GCO), as a complementary testing option integrated within existing sexual health services within British Columbia (BC).	Stakeholder map, desk research, intervies, focus groups, prototypes
Grunberg et al.	2018	[50]	To determine the level of interest in online peer support among infertility patients, factors associated with such interest, and preferences for features of an online peer support network.	Questionnaire
Gude et al.	2012	[51]	Since usability is an important determinant of technology acceptance, we decided to evaluate the usability of PANDIT for both patient and caregiver.	Usability tests
Gulmans et al.	2009	[52]	To evaluate patient care communication in the integrated care setting of children with cerebral palsy in three Dutch regions in order to identify relevant communication gaps experienced by both parents and involved professionals	Desk research, questionnaire, interviews focus group

Harder et al.	2017	[53]	The study aim was to develop a mobile application (app) supported by user preferences to optimise self-management of arm and shoulder exercises for upper-limb dysfunction (ULD) after breast cancer treatment.	Focus groups, literature study, usability tests, requirements,
Hochstenbach et al.	2017	[54]	To describe a co-creative method directed towards the development of na eHealth intervention delivered by registered nurses to support self-management in outpatients with cancer pain.	Literature study, desk research, interviews, focus groups, prototypes, usability tests
Honary et al.	2018	[55]	The aim of this study was to use a user-centered design approach to develop an accessible Web-based intervention, based on the Relatives Education And Coping Toolkit (REACT) booklet, to support the informational and emotional needs of relatives of people experiencing psychosis or bipolar disorder.	Focus groups, requirements, usability tests
Hughes et al.	2020	[56]	This study explored rehabilitation clinician's preferences for design features to be included in an mHealth-enabled app for post-stroke upper limb rehabilitation.	Questionnaire
Imhemed et al.	2017	[57]	To establish the requirements for type-2 diabetes in Malaysia using the user-centered approach.	Questionnaire, focus groups, interviews, prototypes, usability tests
Imhemed et al.	2017	[58]	To leverage user-centered design (UCD), particularly empathy approach to drive the creation of user-personas for type-2 diabetes.	Questionnaire, personas
Jansen et al.	2015	[59]	To explore the perceived need for supportive care including healthy lifestyle programs among cancer survivors, their attitude towards selfmanagement and eHealth, and its association with several sociodemographic and clinical variables and quality of life.	Questionnaire
Jonathan et al.	2021	[60]	This paper describes the user-centered development of LiveWell, a smartphone-based self-management intervention for bipolar disorder, to contribute to and support the ongoing improvement and dissemination of technology-based mental health interventions.	Interviews, focus groups, prototype, usability tests,
Keizer et al.	2020	[61]	This study's aim was to counterbalance the current predominantly top-down, expert-driven audit and feedback approach for antimicrobial resistance prevention measures, with needs and expectations of health care workers.	Interviews
Kelders et al.	2013	[62]	The current study describes the development of a web-based intervention for the indicated prevention of depression, employing the CeHRes roadmap. The goals are to create a user-friendly application which fits the values of the stakeholders and to evaluate the process of development.	Literature study, focus groups, interviews, prototypes, requirements, usability tests
Kelders et al.	2012	[63]	To review the literature on web-based health interventions to investigate whether intervention characteristics and persuasive design affect adherence to a web-based intervention.	Literature study
King et al.	2018	[64]	The purpose of this article is to describe the development and initial usability testing of an eHealth intervention, the Teaching Educators About Chronic Pain Project (TEACh-Pain), designed to increase educator understanding of pediatric chronic pain in the school setting.	Usability tests

Kip et al.	2018	[65]	To examine its potential, this systematic review studies the way that eHealth has been used and studied in forensic mental health and identifies accompanying advantages and disadvantages for both patients and treatment, including effectiveness.	Literature study
Kip et al.	2019	[66]	This study aimed to identify (1) points of improvements in existing forensic mental health treatment of in- and outpatients, (2) possible ways of using VR that can improve current treatment, and (3) positive and negative aspects of the use of VR for the current treatment according to patients and therapists.	Interviews, prototypes, questionnaire,
Kip et al.	2019	[67]	In this paper we show how we formulated values during the multi-method, interdisciplinary and iterative development process of a VR application for a complex setting: forensic mental healthcare	Questionnaire, values, interviews
Kip et al.	2019	[68]	The two main objectives of this case study were to present and reflect on the (1) methods used in the development process of a virtual reality application for forensic mental health care and (2) development model that was used: the CeHRes Roadmap (the Centre for eHealth Research Roadmap).	Stakeholder map, focus groups, literature study, desk research, interviews, prototypes, questionnaire, values,
Kloek et al.	2019	[69]	To develop a blended physiotherapeutic intervention for patients with non-specific low back pain (e-Exercise LBP) and evaluate its proof of concept.	Focus groups, desk research
Kooistra et al.	2016	[70]	To develop a blended cognitive behavioural treatment (bCBT) for depressed patients in an outpatient specialized mental health care centre and to conduct a preliminary evaluation of this bCBT protocol.	Focus groups
Korpershoek et al.	2020	[71]	This study aimed to describe in detail the full user-centred design and development process of an evidence-driven and usable mHealth intervention to enhance exacerbation-related self-management in patients with COPD.	Literature study, Delphi study, interviews, focus groups, behavior change strategies, usability tests, prototype, questionnaire, business model
Kramer et al.	2020	[72]	This review aimed to identify the current practices in designing and evaluating ECAs for coaching people in a healthy lifestyle and provide an overview of their efficacy (on behavioral, knowledge, and motivational parameters) and use (on usability, usage, and user satisfaction parameters).	Literature study
Kristan et al.	2014	[73]	To use online crowdsourcing to evaluate young adult attitudes toward expert-authored messages and to collect peer-authored messages.	Questionnaire
Kulyk et al.	2015	[74]	The ultimate goal of this study is to gain insights into the special needs of this user group, persuasive features and design factors influencing the use and uptake of applications for sexuality, wellbeing and health behavior support of young adults.	Focus groups
Kulyk et al.	2014	[75]	Based on two user studies, we present general guidelines for design and evaluation of lifestyle support systems with personalized virtual coaching. The first field study focuses on design and evaluation of a mobile physical activity coaching system. The second user study focuses on design factors that influence the attitudes of high-risk adolescents towards virtual coaching in mobile eHealth applications and social media.	Questionnaire, interviews, focus group

Kuosmanen et al.	2017	[76]	This study explored student and staff attitudes toward the use of computerized Cognitive Behavioral Therapy in an alternative education setting.	Questionnaire, focus groups
Lambert et al.	2017	[77]	This paper describes the development and theoretical framework for a web-based behavioural activation (BA) intervention, which specifically promotes physical activity (eMotion).	Literature study, focus groups, values, usability tests
Leafman et al.	2018	[78]	To explore potential benefits of TPE and to apply the Community of Inquiry (CoI) framework to examine perceptions of TPE among patients with rare or chronic conditions.	Questionnaire
Lentferink et al.	2017	[79]	To identify key components of self-tracking and persuasive eCoaching in automated healthy lifestyle interventions that contribute to their effectiveness on health outcomes, usability, and adherence. A secondary aim was to identify the way in which these key components should be designed to contribute to improved health outcomes, usability, and adherence.	Literature study
Lentferink et al.	2018	[80]	What are the values according to DSE employees and human resource advisors (HR advisors) for self-tracking, persuasive eCoaching, and preconditions of a workplace stress management application?	Interviews
Lentferink et al.	2020	[81]	This study aims to identify key stakeholders and work with them to gain an in-depth understanding of the value proposition of this stress-management app.	Literature study, questionnaire, interviews, values, stakeholder map, prototype, focus group, requirements
Liao et al.	2017	[82]	The aims of this study were to understand (1) which design features of exercise-promotion apps can enhance quality perception of middle-agers, (2) whether their needs are matched by current functions offered in app stores, and (3) whether physical activity (PA) and mobile phone self-efficacy (MPSE) influence quality perception.	Questionnaire, interviews
Limburg, van et al.	2015	[83]	To demonstrate how business modeling, with the focus on stakeholder involvement, is used to co-create an eHealth implementation.	Literature study, stakeholder map, business model, focus group, interviews
Limburg, van et al.	2015	[84]	This paper focuses on the process of stakeholder involvement to design an implementation strategy for our infectionmanager.com.	Focus group, interviews
Limburg, van et al.	2010	[85]	The following paragraphs describe three recent and different cases of business modeling methods done with the involvement of the authors. These three cases show how the required CDIs were synthesized from different sources in different ways, all using the same business model framework (the business model introduced by Osterwalder) as starting point.	Literature study, interviews, business model, focus group
Liu et al.	2020	[86]	We aimed to develop an RA ‘dashboard’ that could facilitate conversations about PROs and that would be acceptable to a wide range of patients, including English and Spanish speakers and patients with adequate or limited health literacy.	Focus groups, prototypes
Lubberding et al.	2015	[87]	To gain insight into cancer survivors’ needs towards an eHealth application monitoring quality of life and targeting personalised access to supportive care.	Interviews

Maguire et al.	2015	[88]	This study aimed to (a) explore the feasibility and acceptability of the Advanced Symptom Management System with patients with lung cancer receiving radiotherapy and clinicians involved in their care and (b) assess changes in patient outcomes during implementation of the Advanced Symptom Management System with patients with lung cancer receiving radiotherapy in clinical practice.	Literature study, focus groups
Marshall et al.	2019	[89]	The present study forms part of a larger project, whose ultimate aim is to develop digital technology to support outcomes for LBP.	Interviews
McGrath et al.	2017	[90]	In order to help extend coverage to more rural student athletes, we propose designing a telemedicine app following the mHealth development roadmap from the Center for eHealth Research (CeHRes). In this paper we will document contextual inquiry, user requirements capture, design phases, and app evaluation from the targeted user base.	Stakeholder map, literature study, focus groups, prototype, questionnaire
Meiland et al.	2017	[91]	The aim of our study was to review the state of the art of technologies for persons with dementia regarding issues on development, usability, effectiveness and cost-effectiveness, deployment, and ethics in 3 fields of application of technologies: (1) support with managing everyday life, (2) support with participating in pleasurable and meaningful activities, and (3) support with dementia health and social care provision.	Literature study
Mirk et al.	2017	[92]	Regarding a mobile app for patient education, the objectives of the study were to (1) quantify patient interest, (2) determine desirable features, and (3) determine if a relationship exists between patient variables and interest in an iPad app for patient education.	Questionnaire
Mummah et al.	2016	[93]	This research sought to iteratively develop a theory-driven mobile app, Vegethon, to increase vegetable consumption.	Interviews, literature study, behavior change strategies, focus group, prototypes, questionnaires
Nicklas et al.	2020	[94]	We sought to employ an interactive, user-centered, and participatory method of development, evaluation, and iteration to design and optimize the mobile health (mHealth) Fit After Baby program.	Questionnaire, log data analysis, interviews
Nijland et al.	2010	[95]	We studied a web-based triage system which was accessible to the general public in the Netherlands. In a etrospective analysis we investigated the type of complaints that were submitted and the kind of advice provided.	Log data analysis, questionnaire
Nobakht et al.	2018	[96]	To develop a user-friendly web based intervention for training parents of children with CP and evaluate the process of development using modified CeHRes roadmap.	Interviews, questionnaire, prototype, focus group
Noordman et al.	2019	[97]	This study aims to evaluate the usability, perceived usefulness, and actual use of ListeningTime, through the eyes of elderly patients with cancer and their oncological HCPs.	Questionnaires, log data analysis
Noordman et al.	2017	[98]	To develop ListeningTime in a participatory way to increase uptake and use.	Intervies, focus groups, prototype, usability tests
Norris et al.	2015	[99]	This paper describes the initial stages of a project to develop such a paradigm by scoping and developing the area of disaster e-health.	Literature study
Oberschmidt et al.	2020	[100]	In this article, we report on a co-design process with different stakeholders and the negotiation that ensued after the elicitation of their wishes.	Focus groups, prototypes

Organ et al.	2018	[101]	With concern growing about the capacity of mHealth interventions to engage users in real world settings, we undertook a systematic review of the implementation and reporting of user-centred design (UCD) practices in the development of illicit substance use behavioural interventions in the higher education context (n=7).	Literature study
Ossebaard et al.	2012	[102]	First objective is to examine what usability aspects of the portal kiesBeter.nl matter for chronic patients and their informal carers with regard to information seeking, self-management, decision making, on line health information and other variables. Second objective is to make evidence-based practical recommendations for usability improvement.	Questionnaire, interviews, usability tests, focus group,
Pang et al.	2016	[103]	The aim of this research was to create a design for consumer health websites by supporting different health information–seeking behaviors. We created a website called Better Health Explorer with the new design. Through the evaluation of this new design, we derive design implications for future implementations.	Log data analysis, interviews
Pearson et al.	2016	[104]	The objective of this study was to codesign a Web-based version of ESCAPE-pain that people with chronic joint pain find engaging, informative, and useful.	Questionnaire, focus groups, interviews, prototype, usability test
Radomski et al.	2019	[105]	The objectives of this study were to use a realist synthesis approach to explore the design and delivery features of iCBT for children and adolescents with anxiety as described in the literature and to examine their relationship to program use outcomes.	Literature study
Ragouzeos et al.	2019	[106]	We aimed to develop a "dashboard" for RA patients to display relevant PRO measures for discussion during a routine RA clinical visit.	Interviews, focus groups, usability tests
Rai et al.	2020	[107]	This study aims to develop and investigate the usability of Thinkability, an Individual Cognitive Stimulation Therapy (iCST) app that can be used by people with dementia and carers on touchscreen tablets.	Focus groups, desk research, prototypes, interviews
Rai et al.	2020	[108]	To test the individual Cognitive Stimulation Therapy (iCST) application with people with dementia and carers in order to modify and refine the application, and improve its usability.	Focus groups, interviews, questionnaire
Reblin et al.	2017	[109]	The aim of our study was to provide an example of the application of the Web-based intervention development process using the CeHRes Roadmap for other research teams to follow. In doing so, we are also sharing our pilot work to enhance eSNAP's acceptance and usability for users and the feasibility of its implementation.	Literature study, interviews, focus groups, prototypes
Rosen, von et al.	2017	[110]	To assess students' preferences when looking for sexual health information online.	Questionnaire
Rothgangel et al.	2017	[111]	To describe the user-centered approach that guided the design and development of a telerehabilitation platform for patients with phantom limb pain.	Questionnaire, interviews, literature study, prototypes
Schmidt et al.	2021	[112]	To design and evaluate a mental health treatment program and internet-based delivery platform for patients with ischemic heart disease (IHD) attending cardiac rehabilitation with the aim of reducing the risks associated with anxiety and/or depression.	Focus groups
Scholten et al.	2019	[113]	Can we realize user experience improvements on a text- and video-based self-guided e-mental health intervention by adding an ECA that makes users more engaged and motivated? The aim of this study is to investigate the opportunities from within the technology to increase its support level toward the user.	Questionnaire

Scholten et al.	2017	[114]	This study aimed to explore supportive capabilities of virtual agents with the potential benefit in mind that users of self-guided eHealth interventions could be better supported.	Literature study
Scholten et al.	2017	[115]	The first objective of this paper was to explore what is known in literature about what support a user needs to stay motivated and engaged in an electronic health (eHealth) intervention that requires repeated use. The second objective was to explore the current potential of embodied conversational agents (ECAs) to provide this support.	Literature study
Sewitch et al.	2019	[116]	The aim of this qualitative study was to employ a user-centered approach to design the content and features of a smartphone app called colonAPPscopy to support individuals preparing for their colonoscopy appointments.	Focus groups
Sieverink et al.	2017	[117]	To determine the extent to which a participatory development process can be supportive for developing human-centered eHealth technologies as opposed to expert-driven information systems (to improve education for MRSA-carriers in the home situation by developing a web-based communication system that meets both cognitive (practical) and affective (personal) information needs and to provide healthcare providers with information that is easy to access and to use).	Log data analysis
Sjölinder et al.	2014	[118]	This work describes a method used in the development of a stroke rehabilitation tool.	Focus groups
Skjoth et al.	2015	[119]	The aim of this study was to develop an eHealth tool that contained accurate and relevant information to allow pregnant women to make an informed choice about whether to accept or reject participation in screening for Down syndrome.	Literature review, focus groups, interviews
Solem et al.	2020	[120]	To design and develop a user-centered, evidence-based eHealth self-management intervention for people with chronic pain.	Desk research, personas, focus groups, prototypes, usability tests
Span et al.	2015	[121]	The aim of this study is to identify design issues in developing a user-friendly IT application facilitating Shared decision-making in dementia.	Focus groups, usability tests, prototypes
Span et al.	2014	[122]	The aim of this study was to gain insight in the user friendliness of the DecideGuide, user acceptance and satisfaction, and participants' opinion of the DecideGuide for making decisions	Interviews, log data analysis,
Span et al.	2014	[123]	The aim of this study is to identify design issues (both weaknesses and strengths) that should be considered for designing a user-friendly design of an interactive web tool that facilitates shares decision making in care networks of people with dementia.	Focus groups, prototypes, usability tests
Span et al.	2014	[124]	To identify user requirements for an interactive web tool for people with dementia, informal caregivers, case managers and other professional caregivers	Interviews, focus groups
Spanakis et al.	2016	[125]	To identify and discuss the main scientific and engineering challenges that need to be successfully addressed in delivering state-of-the-art, ubiquitous eHealth and mHealth services, including citizen-centered wellness management services, and reposition their role and potential within a broader context of diverse sociotechnical drivers, agents, and stakeholders.	Literature study
Sporrel et al.	2021	[126]	In this paper, we describe the design and development of the Playful data-driven Active Urban Living (PAUL): a personalized physical activity application.	Interviews, requirements, behavior change strategies, desk research, focus groups,

Stara et al.	2020	[127]	This systematic literature review aimed to identify and synthesize published literature focusing on the efficacy of digital health coaching interventions specifically designed for older workers in transition to retirement (or those who just retired) and that used the user-centered design approach.	Literature study
Stuij et al.	2020	[128]	We describe the design of a digital communication skills training (CST)-tool for information provision skills that meets oncologists' learning needs. We used the CeHRes Roadmap for user-centred design as a guiding framework.	Literature study, focus groups, usability tests, prototype, requirements
Stuij et al.	2018	[129]	1) uncover the learning needs of oncological healthcare providers related to information- provision, and 2) explore their training preferences in the context of clinical practice.	Focus groups, interviews
Suffoletto et al.	2017	[130]	To explore whether a text message-based relapse prevention intervention (Preventing and Interrupting Early Relapse [PIER]1) is acceptable to treatment-seeking adults with opioid use disorder (OUD) after Emergency Department (ED) discharge using mixed-methods design.	Interviews, log data analysis
Teles et al.	2017	[131]	Which are the factors supporting and hindering the development and, in the ongoing process, the implementation of the ActiveAdvice platform, regarding user requirements, preferences, acceptances and expectations?	Interviews
Tieman et al.	2014	[132]	This study examined the processes involved in developing a prototype telehealth intervention to support palliative care patients involved with a palliative care service living in the community.	Focus groups, prototype
Timmer et al.	2020	[133]	The aim of this study was to develop a blended physiotherapy intervention for persons with hemophilic arthropathy.	Literature study, focus groups,
Timmerman et al.	2016	[134]	1) to develop a multimodal telehealthcare application that aimed at improving post-surgery rehabilitation and physical activity, in close cooperation with resected NSCLC survivors and their healthcare professionals (HCPs), and 2) to evaluate its usability.	Interviews, focus groups, usability tests, prototypes
Uden-Kraan, van et al.	2020	[135]	To investigate among patients treated with a total laryngectomy (TL) (1) Internet-use and Internet use to search for information on health and cancer (content); (2) which patients are most likely to use the Internet in general, for health-related and cancer-related purposes; (3) which other types of eHealth (community, communication, care) are used; and (4) preferences towards future use.	Questionnaire
Vaart, van der et al.	2014	[136]	The aim of this study was to gain (1) insight into which treatment components are most suitable for online and/or face-to-face sessions, (2) the sequence and ratio the two forms of therapy should be combined in, and (3) the essential points of interest that should be considered when implementing blended care for depression in secondary mental health care (i.e. health care services provided by psychiatrists or clinical psychologists).	Questionnaires, interviews, Delphi study
Velsen, van et al.	2015	[137]	Discusses the development of requirements for an mHealth intervention aimed at supporting citizens to deal with ticks and tick bites and persuading them to comply with preventive advice.	Interviews, focus groups, requirements
Velsen, van et al.	2014	[138]	To assess the general public's perceptions, knowledge, preventive behavior and sources of information (to answer the questions Which information should health organizations convey during a largescale Salmonella outbreak, and by which channels, to maximize citizen compliance with preventive advice?)	Questionnaire
Velsen, van et al.	2012	[139]	The goal of our study was to assess citizens' Web 2.0 media use during an infectious disease outbreak and to determine which Web 2.0 medium is used for which goal. With this information, we wanted to formulate	Experience sampling, questionnaire

			recommendations for health organizations that consider using Web 2.0 media as part of their communication strategy during an infectious disease outbreak.	
Verhoeven et al.	2010	[140]	To determine the effects of teleconsultation regarding clinical, behavioral, and care coordination outcomes of diabetes care compared to usual care.	Literature study
Verhoeven et al.	2010	[141]	With this study, we aim to identify a discount approach for user-centered design of a completely new e-health application (without a prototype being available).	Usability tests, card sorting, prototypes,
Verhoeven et al.	2010	[142]	In order to enhance the usability of the expert-driven guideline format, we developed a website for the communication of existing guidelines that better fit the practical information needs of health care workers (HCWs).	Usability tests, requirements,
Verhoeven et al.	2010	[143]	To assess nurses' information-seeking strategies and problems encountered when seeking clinical guidelines on the Internet, and to investigate the criteria they apply when evaluating the guidelines and the websites communicating the guidelines.	Usability tests
Verhoeven et al.	2009	[144]	To describe the design process of a web-based tool for compliance with safe work practices in cross-border care settings, focusing on infection control of MRSA.	Desk research, questionnaire, focus group, card sorting, prototypes, interviews
Verhoeven et al.	2008	[145]	This paper describes the steps that appeared to be crucial for the development of a tool that aims to provide HCWs with acceptable, applicable, user-friendly and high quality MRSA guidelines that enhance the decision making process to deliver safe health care.	Desk research, usability test, questionnaire, prototype, interviews, card sorting
Verhoeven et al.	2007	[146]	To determine the benefits and deficiencies of teleconsultation and videoconferencing regarding clinical, behavioral, and care coordination outcomes of diabetes care.	Literature study
Vermeulen et al.	2013	[147]	To involve elderly people during the development of a mobile interface of a monitoring system that provides feedback to them regarding changes in physical functioning and to test the system in a pilot study.	Literature study, focus groups, usability tests, prototypes
Wachtler et al.	2018	[148]	In this study, we describe the user-centered design process of an app to assess individual risk of persistent depressive symptoms and recommend individually tailored treatment based on current knowledge about best-evidence treatment for depression.	Focus groups, interviews, prototype, desk research
Wahle et al.	2017	[149]	Updating prior reviews, this study aims to (1) assess the effectiveness of technology-supported interventions for the treatment of depression and (2) add to the debate on what components in technology-mediated MHIS for the treatment of depression should be standard of care.	Literature study
Walters et al.	2018	[150]	To identify and define design requirements for the development of a teledermatology scale-up framework (TDSF)	Interviews, literature study, requirements
Walters et al.	2016	[151]	This study reports on the current status of teledermatology services in the public health sector of SA.	Literature study
Wentink et al.	2019	[152]	To identify end-user requirements for a comprehensive eHealth program in stroke rehabilitation.	Focus groups, interviews
Wentink et al.	2018	[153]	To prioritize the requirements for stroke e-rehabilitation according to patients, informal caregivers, and health professionals.	Questionnaire

Wentzel et al.	2016	[154]	We describe postulates for blended care and provide an instrument (Fit for Blended Care) that aims to assist therapists and patients whether and how to set up blended care treatment.	Literature study, focus groups, interviews
Wentzel et al.	2014	[155]	In this formative study, we investigate how nurses' can be supported in antimicrobial stewardship by means of an eHealth intervention that targets their information needs.	Focus group, card sorting, desk research, usability tests
Wentzel et al.	2011	[156]	In sum, we apply the CeHRes roadmap by starting with a summative evaluation of MRSA-net. This evaluation also serves as input for the contextual inquiry phase of Eursafety Health Net and renders input for the eDEHN-platform.	Log data analysis
Whiteside et al.	2014	[157]	The aim of this study was to get user input and feedback on acceptability of messaging content intended to engage suicidal individuals.	Questionnaire
Winterling et al.	2016	[158]	The study aims to describe the development of a Web-based intervention in long-term collaboration with patient research partners (PRPs).	Focus groups, prototypes
Woezik, van et al.	2016	[159]	To develop a guideline to assist developers in tackling wicked problems using co-creation with stakeholders, and to apply this guideline to practice with an example case in the field of infection prevention and control.	Literature study, questionnaire, interviews,
Yap et al.	2017	[160]	In this paper, we propose a multi-level public health approach involving a Web-based parenting intervention, Partners in Parenting (PIP). We describe the components of the Web-based intervention and how each component was developed.	Literature study, Delphi study, focus groups, usability tests, prototype

1. Al-Itejawi, H.H.M., et al., *Development of a patient decision aid for the treatment of localised prostate cancer: a participatory design approach.* Journal of Clinical Nursing, 2016. **25**(7-8): p. 1131-1144.
2. Amann, J., et al., *Opportunities and Challenges of a Self-Management App to Support People With Spinal Cord Injury in the Prevention of Pressure Injuries: Qualitative Study.* Jmir Mhealth and Uhealth, 2020. **8**(12).
3. Ammerlaan, J.W., et al., *Building a Tailored, Patient-Guided, Web-Based Self-Management Intervention 'ReumaUitgedaagd!' for Adults With a Rheumatic Disease: Results of a Usability Study and Design for a Randomized Control Trail.* Jmir Research Protocols, 2016. **5**(2).
4. Asbjørnsen, R.A., et al., *Persuasive system design principles and behavior change techniques to stimulate motivation and adherence in electronic health interventions to support weight loss maintenance: Scoping review.* Journal of Medical Internet Research, 2019. **21**(6).
5. Asbjornsen, R.A., et al., *Identifying Persuasive Design Principles and Behavior Change Techniques Supporting End User Values and Needs in eHealth Interventions for Long-Term Weight Loss Maintenance: Qualitative Study.* Journal of Medical Internet Research, 2020. **22**(11).
6. Askedal, K. and L.S. Flak, *Stakeholder Contradictions in Early Stages of eHealth Efforts.* … of the 50th …, 2017.
7. De Backere, F., et al., *The OCareCloudS project: Toward organizing care through trusted cloud services.* Informatics for Health & Social Care, 2016. **41**(2): p. 159-176.
8. Beaujean, D., et al., *Using risk group profiles as a lightweight qualitative approach for intervention development: An example of prevention of tick bites and lyme disease.* Journal of Medical Internet Research, 2013. **15**(10).

9. Beerlage-de Jong, N., et al., *Combining user-centered design with the persuasive systems design model: The development process of a web-based registration and monitoring system for healthcare-associated infections in nursing homes.* International Journal on Advances in Life Sciences, 2014. **6**(3-4): p. 262-271.
10. Beerlage-de Jong, N., et al., *The value of participatory development to support antimicrobial stewardship with a clinical decision support system.* American Journal of Infection Control, 2017. **45**(4): p. 365-371.
11. Jong, N.B.-d. and N. Köhle, *Van 'vreemde vogels' en vogelgriep.* Infectieziekten …, 2017.
12. Beerlage-de Jong, N., et al., *Technology to support integrated Antimicrobial Stewardship Programs: a user centered and stakeholder driven development approach.* Infectious disease reports, 2017. **9**(1).
13. Van de Belt, T.H., et al., *Internet and Social Media For Health-Related Information and Communication in Health Care: Preferences of the Dutch General Population.* Journal of Medical Internet Research, 2013. **15**(10): p. 152-162.
14. Bernecker, S.L., et al., *A Web-Disseminated Self-Help and Peer Support Program Could Fill Gaps in Mental Health Care: Lessons From a Consumer Survey.* Jmir Mental Health, 2017. **4**(1).
15. Boele, F.W., et al., *Neuro-oncology family caregivers' view on keeping track of care issues using eHealth systems: it's a question of time.* Journal of Neuro-Oncology, 2017. **134**(1): p. 157-167.
16. Bogza, L.M., et al., *User-Centered Design and Evaluation of a Web-Based Decision Aid for Older Adults Living With Mild Cognitive Impairment and Their Health Care Providers: Mixed Methods Study.* Journal of Medical Internet Research, 2020. **22**(8).
17. Børøsund, E., et al., *A stress management app intervention for cancer survivors: Design, development, and usability testing.* Journal of Medical Internet Research, 2018. **20**(9).
18. Bossen, D., et al., *A Blended Intervention for Patients With Knee and Hip Osteoarthritis in the Physical Therapy Practice: Development and a Pilot Study.* Jmir Research Protocols, 2016. **5**(1).
19. Breeman, L.D., et al., *A multi-stakeholder approach to eHealth development: Promoting sustained healthy living among cardiovascular patients.* International Journal of Medical Informatics, 2021. **147**.
20. van Bruinessen, I.R., et al., *Active patient participation in the development of an online intervention.* JMIR Res Protoc, 2014. **3**(4): p. e59.
21. Carr, E.C., J.N. Babione, and D. Marshall, *Translating research into practice through user-centered design: an application for osteoarthritis healthcare planning.* International journal of medical informatics, 2017. **104**: p. 31-37.
22. Cheung, K.L., et al., *Optimizing Usability of an Economic Decision Support Tool: Prototype of the Equipt Tool.* Int J Technol Assess Health Care, 2018. **34**(1): p. 68-77.
23. Cnossen, I.C., et al., *A Participatory Design Approach to Develop a Web-Based Self-Care Program Supporting Early Rehabilitation among Patients after Total Laryngectomy.* Folia Phoniatrica Et Logopaedica, 2015. **67**(4): p. 193-201.
24. Corbett, T., et al., *Cancer-Related Fatigue in Post-Treatment Cancer Survivors: Theory-Based Development of a Web-Based Intervention.* Jmir Cancer, 2017. **3**(2).
25. Crane, D., et al., *Factors Influencing Usability of a Smartphone App to Reduce Excessive Alcohol Consumption: Think Aloud and Interview Studies.* Frontiers in Public Health, 2017. **5**.

26. Cruz-Martínez, R.R., et al., *Towards data-driven persuasive coaching in a heart failure telemonitoring technology.* CEUR Workshop Proceedings, 2018. **2102**: p. 60-75.
27. Da Costa, D., et al., *HealthyDads. ca: What Do Men Want in a Website Designed to Promote Emotional Wellness and Healthy Behaviors During the Transition to Parenthood?* Journal of Medical Internet Research, 2017. **19**(10).
28. D'Addario, M., et al., *Engagement Features in Physical Activity Smartphone Apps: Focus Group Study With Sedentary People.* Jmir Mhealth and Uhealth, 2020. **8**(11).
29. Derks, Y. and T.D. Visser, *mHealth in mental health: how to efficiently and scientifically create an ambulatory biofeedback e-coaching app for patients with borderline personality disorder.* … Journal of Human …, 2017.
30. Derks, Y.P., et al., *Development of an Ambulatory Biofeedback App to Enhance Emotional Awareness in Patients with Borderline Personality Disorder: Multicycle Usability Testing Study.* JMIR mHealth and uHealth, 2019. **7**(10): p. e13479.
31. Dick, S., C. Heavin, and V.S. Vasiliou, *The Design of a Digital Behaviour Change Intervention for Third-Level Student Illicit Substance Use: A Persona Building Approach.* … of the 53rd …, 2020.
32. Van De Dijk, M., et al., *ECOPD: User requirements of older people with copd for ehealth support at home, a user-centred study.* 2013. **33**: p. 1272-1278.
33. Doty, J.L., et al., *Designing a Mobile App to Enhance Parenting Skills of Latinx Parents: A Community-Based Participatory Approach.* JMIR Form Res, 2020. **4**(1): p. e12618.
34. Downing, K.L., et al., *Informing Active Play and Screen Time Behaviour Change Interventions for Low Socioeconomic Position Mothers of Young Children: What Do Mothers Want?* Biomed Research International, 2016.
35. Duman-Lubberding, S., et al., *An eHealth Application in Head and Neck Cancer Survivorship Care: Health Care Professionals' Perspectives.* Journal of Medical Internet Research, 2015. **17**(10).
36. Duplaga, M., *Searching for a role of nursing personnel in developing landscape of ehealth: Factors determining attitudes toward key patient empowering applications.* PLoS ONE, 2016. **11**(4).
37. Van Dyk, L., et al., *Business models for sustained ehealth implementation: Lessons from two continents.* 2012. **2**: p. 889-904.
38. Ehrler, F., C. Lovis, and K. Blondon, *A Mobile Phone App for Bedside Nursing Care: Design and Development Using an Adapted Software Development Life Cycle Model.* Jmir Mhealth and Uhealth, 2019. **7**(4).
39. Fico, G., M. Teresa Arredondo, and Ieee, *Use of an Holistic Approach for effective adoption of User-Centred-Design Techniques in Diabetes Disease Management: experiences in User Need Elicitation*, in *2015 37th Annual International Conference of the Ieee Engineering in Medicine and Biology Society*. 2015. p. 2139-2142.
40. Fico, G., et al., *User Centered Design to incorporate predictive models for Type 2 Diabetes screening and management into professional decision support tools: preliminary results*, in *World Congress on Medical Physics and Biomedical Engineering, 2015, Vols 1 and 2*, D.A. Jaffray, Editor. 2015. p. 1389-1392.
41. Fico, G., et al., *What do healthcare professionals need to turn risk models for type 2 diabetes into usable computerized clinical decision support systems? Lessons learned from the MOSAIC project.* Bmc Medical Informatics and Decision Making, 2019. **19**(1).

42. Fledderus, M., et al., *Development and Pilot Evaluation of an Online Relapse-Prevention Program Based on Acceptance and Commitment Therapy for Chronic Pain Patients.* Journal of Medical Internet Research, 2015. **17**(1).
43. Floch, J., et al., *User needs in the development of a health app ecosystem for self-management of cystic fibrosis: User-centered development approach.* JMIR mHealth and uHealth, 2018. **6**(5).
44. Galpin, A., et al., *"Thanks for Letting Us All Share Your Mammogram Experience Virtually": Developing a Web-Based Hub for Breast Cancer Screening.* Jmir Cancer, 2017. **3**(2).
45. van Gemert-Pijnen, L. and M. Span, *CeHRes roadmap to improve dementia care.* 2016: p. 133-146.
46. van Gemert-Pijnen, L., et al., *Participatory development via user-involvement - A case study about the development of a Web-based patient-communication system about methicillin-resistant staphylococcus aureus.* Electronic Journal of Health Informatics, 2011. **6**(4).
47. Van Gemert-Pijnen, J.E.W.C., S.M. Kelders, and E.T. Bohlmeijer, *Understanding the usage of content in a mental health intervention for depression: An analysis of log data.* Journal of Medical Internet Research, 2014. **16**(1).
48. Geraghty, A.W.A., et al., *Developing an Unguided Internet-Delivered Intervention for Emotional Distress in Primary Care Patients: Applying Common Factor and Person-Based Approaches.* Jmir Mental Health, 2016. **3**(4).
49. Gilbert, M., et al., *Get checked… where? the development of a comprehensive, integrated internet-based testing program for sexually transmitted and blood-borne infections in ….* JMIR research …, 2016.
50. Grunberg, P.H., et al., *Infertility patients' need and preferences for online peer support.* Reprod Biomed Soc Online, 2018. **6**: p. 80-89.
51. Gude, W.T., et al., *Formative usability evaluation of a web-based insulin self-titration system: preliminary results.* Stud Health Technol Inform, 2012. **180**: p. 1209-11.
52. Gulmans, J., et al., *Evaluating patient care communication in integrated care settings: Application of a mixed method approach in cerebral palsy programs.* International Journal for Quality in Health Care, 2009. **21**(1): p. 58-65.
53. Harder, H., et al., *A user-centred approach to developing bWell, a mobile app for arm and shoulder exercises after breast cancer treatment.* Journal of Cancer Survivorship, 2017. **11**(6): p. 732-742.
54. Hochstenbach, L.M., et al., *Co-creative development of an eHealth nursing intervention: Self-management support for outpatients with cancer pain.* Applied Nursing Research, 2017. **36**: p. 1-8.
55. Honary, M., et al., *A Web-Based Intervention for Relatives of People Experiencing Psychosis or Bipolar Disorder: Design Study Using a User-Centered Approach.* Jmir Mental Health, 2018. **5**(4).
56. Hughes, C.M.L., et al., *Developing an mHealth app for post-stroke upper limb rehabilitation: Feedback from US and Ethiopian rehabilitation clinicians.* Health Informatics Journal, 2020. **26**(2): p. 1104-1117.
57. Imhemed, S.M., A. Kamaruddin, and S. Hasan, *Designing a Mobile App to support a Healthy Lifestyle (MAS-HeaL) for type-2 diabetes patients in Malaysia.* Journal of Engineering and Applied Sciences, 2017. **12**(3): p. 559-563.
58. IMHEMED, S.M., A. KAMARUDDIN, and R. ATAN, *Using Empathy Approach to Design Type-2 Diabetic User Persona.*
59. Jansen, F., et al., *Cancer survivors' perceived need for supportive care and their attitude towards self-management and eHealth.* Supportive Care in Cancer, 2015. **23**(6): p. 1679-1688.

60. Jonathan, G.K., et al., *A Smartphone-Based Self-management Intervention for Bipolar Disorder (LiveWell): User-Centered Development Approach.* Jmir Mental Health, 2021. **8**(4).
61. Keizer, J., et al., *Finding the match between healthcare worker and expert for optimal audit and feedback on antimicrobial resistance prevention measures.* Antimicrobial Resistance & Infection Control, 2020. **9**(1): p. 1-12.
62. Kelders, S.M., et al., *Development of a web-based intervention for the indicated prevention of depression.* BMC Medical Informatics and Decision Making, 2013. **13**(1).
63. Kelders, S.M., et al., *Persuasive system design does matter: A systematic review of adherence to web-based interventions.* Journal of Medical Internet Research, 2012. **14**(6).
64. King, S. and J.A. Boutilier, *Managing Chronic Pain in the Classroom: Development and Usability Testing of an eHealth Educational Intervention for Educators.* Canadian Journal of …, 2018.
65. Kip, H., et al., *eHealth in treatment of offenders in forensic mental health: A review of the current state.* Frontiers in Psychiatry, 2018. **9**(FEB).
66. Kip, H., et al., *Identifying the Added Value of Virtual Reality for Treatment in Forensic Mental Health: A Scenario-Based, Qualitative Approach.* Frontiers in Psychology, 2019. **10**.
67. Kip, H., et al. *Putting the Value in VR How to Systematically and Iteratively Develop a Value-Based VR Application with a Complex Target Group*. in *CHIConference on Human Factors in Computing Systems Proceedings (CHI2019),*. 2019. Glasgow, Scotland Uk: ACM.
68. Kip, H., S.M. Kelders, and Y.H.A. Bouman, *The importance of systematically reporting and reflecting on eHealth development: Participatory development process of a virtual reality application for ….* Journal of medical …, 2019.
69. Kloek, C.J.J., et al., *Development and proof of concept of a blended physiotherapeutic intervention for patients with non-specific low back pain.* Physiotherapy, 2019. **105**(4): p. 483-491.
70. Kooistra, L.C., et al., *Development and initial evaluation of blended cognitive behavioural treatment for major depression in routine specialized mental health care.* Internet Interventions, 2016. **4**: p. 61-71.
71. Korpershoek, Y.J.G., et al., *User-Centered Design of a Mobile Health Intervention to Enhance Exacerbation-Related Self-Management in Patients With Chronic Obstructive Pulmonary Disease (Copilot): Mixed Methods Study.* Journal of Medical Internet Research, 2020. **22**(6).
72. Kramer, L.L., et al., *Developing Embodied Conversational Agents for Coaching People in a Healthy Lifestyle: Scoping Review.* Journal of Medical Internet Research, 2020. **22**(2).
73. Kristan, J. and B. Suffoletto, *Using online crowdsourcing to understand young adult attitudes toward expert-authored messages aimed at reducing hazardous alcohol consumption and to collect peer-authored messages.* Translational Behavioral Medicine, 2015. **5**(1): p. 45-52.
74. Kulyk, O., et al., *How persuasive are serious games, social media and mHealth technologies for vulnerable young adults? Design factors for health behavior and lifestyle change support: Sexual health case.* CEUR Workshop Proceedings, 2015. **1369**: p. 28-42.
75. Kulyk, O., et al., *Personalized virtual coaching for lifestyle support: Principles for design and evaluation.* International Journal on Advances in Life Sciences, 2014. **6**(3-4): p. 300-309.
76. Kuosmanen, T., T.M. Fleming, and M.M. Barry, *Using Computerized Mental Health Programs in Alternative Education: Understanding the Requirements of Students and Staff.* Health Communication, 2018. **33**(6): p. 753-761.

77. Lambert, J.D., et al., *Development of a web-based intervention (eMotion) based on behavioural activation to promote physical activity in people with depression.* Mental Health and Physical Activity, 2017. **13**: p. 120-136.
78. Leafman, J.S. and K. Mathieson, *Perceptions of telemedicine for patient education among online support group patients with chronic or rare conditions.* Cogent Medicine, 2018.
79. Lentferink, A.J., et al., *Key components in ehealth interventions combining self-tracking and persuasive eCoaching to promote a healthier lifestyle: A scoping review.* Journal of Medical Internet Research, 2017. **19**(8).
80. Lentferink, A., et al., *The Values of Self-tracking and Persuasive eCoaching According to Employees and Human Resource Advisors for a Workplace Stress Management Application: A Qualitative Study.* 2018. **10809 LNCS**: p. 160-171.
81. Lentferink, A., et al., *Creating value with eHealth: identification of the value proposition with key stakeholders for the resilience navigator app.* Bmc Medical Informatics and Decision Making, 2020. **20**(1).
82. Liao, G.-Y., et al., *What to Build for Middle-Agers to Come? Attractive and Necessary Functions of Exercise-Promotion Mobile Phone Apps: A Cross-Sectional Study.* Jmir Mhealth and Uhealth, 2017. **5**(5).
83. van Limburg, M., et al., *Business Modeling to Implement an eHealth Portal for Infection Control: A Reflection on Co-Creation With Stakeholders.* Jmir Research Protocols, 2015. **4**(3).
84. Limburg, M.v., H.C. Ossebaard, and M.J. Wentzel, *Co-creating with stakeholders: ideating eHealth applications to support antibiotic stewardship in hospitals.* 2015.
85. Van Limburg, A.H.M. and J. Van Gemert-Pijnen, *Towards innovative business modeling for sustainable eHealth applications.* 2nd International Conference on eHealth, Telemedicine, and Social Medicine, eTELEMED 2010, Includes MLMB 2010; BUSMMed 2010, 2010: p. 11-16.
86. Liu, L.H., et al., *Patient and clinician perspectives on a patient-facing dashboard that visualizes patient reported outcomes in rheumatoid arthritis.* Health Expectations, 2020. **23**(4): p. 846-859.
87. Lubberding, S., et al., *Improving access to supportive cancer care through an eHealth application: A qualitative needs assessment among cancer survivors.* Journal of Clinical Nursing, 2015. **24**(9-10): p. 1367-1379.
88. Maguire, R., et al., *Development of a Novel Remote Patient Monitoring System The Advanced Symptom Management System for Radiotherapy to Improve the Symptom Experience of Patients With Lung Cancer Receiving Radiotherapy.* Cancer Nursing, 2015. **38**(2): p. E37-E47.
89. Marshall, C.J., et al., *User-centered value specifications for technologies supporting chronic low-back pain management*, in *Studies in Health Technology and Informatics*. 2019. p. 1288-1292.
90. McGrath, S.P., M.L. McGrath, and D. Bastola, *Developing a concussion assessment mHealth app for certified Athletic Trainers.* AMIA Annu Symp Proc, 2017. **2017**: p. 1282-1291.
91. Meiland, F., et al., *Technologies to support community-dwelling persons with dementia: a position paper on issues regarding development, usability, effectiveness and cost ….* JMIR rehabilitation …, 2017.
92. Mirk, S.M. and N.M. Wegrzyn, *Apps for health-related education in pharmacy practice: needs assessment survey among patients within a large metropolitan area.* JMIR research protocols, 2017.
93. Mummah, S.A., et al., *Iterative development of Vegethon: a theory-based mobile app intervention to increase vegetable consumption.* International Journal of Behavioral Nutrition and Physical Activity, 2016. **13**.

94. Nicklas, J.M., et al., *Development and modification of a mobile health program to promote postpartum weight loss in women at elevated risk for cardiometabolic disease: single-arm pilot study.* JMIR Formative Research, 2020. **4**(4).
95. Nijland, N., et al., *Patient use and compliance with medical advice delivered by a web-based triage system in primary care.* Journal of Telemedicine and Telecare, 2010. **16**(1): p. 8-11.
96. Nobakht, Z., M. Rassafiani, and S.A. Hosseini, *A Web-Based Caring Training for Caregivers of Children with Cerebral Palsy: Development and Evaluation.* Iran J Child Neurol, 2018. **12**(4): p. 65-84.
97. Noordman, J., et al., *Evaluation and Implementation of ListeningTime: A Web-Based Preparatory Communication Tool for Elderly Patients With Cancer and Their Health Care Providers.* Jmir Cancer, 2019. **5**(1).
98. Noordman, J., et al., *ListeningTime; participatory development of a web-based preparatory communication tool for elderly cancer patients and their healthcare providers*. 2017.
99. Norris, A.C., et al., *Disaster E-Health: A New Paradigm for Collaborative Healthcare in Disasters.* ISCRAM, 2015.
100. Oberschmidt, K., et al. *You can't always get what you want: Streamlining stakeholder interests when designing technology-supported services for Active and Assisted Living*. in *ACM International Conference Proceeding Series*. 2020.
101. Organ, D., et al., *A systematic review of user-centred design practices in illicit substance use interventions for higher education students.* … on Information Systems …, 2018.
102. Ossebaard, H.C., E.R. Seydel, and L. van Gemert-Pijnen, *Online usability and patients with long-term conditions: A mixed-methods approach.* International Journal of Medical Informatics, 2012. **81**(6): p. 374-387.
103. Pang, P.C.-I., et al., *Designing Health Websites Based on Users' Web-Based Information-Seeking Behaviors: A Mixed-Method Observational Study.* Journal of Medical Internet Research, 2016. **18**(6).
104. Pearson, J., et al., *Developing a Web-Based Version of An Exercise-Based Rehabilitation Program for People With Chronic Knee and Hip Pain: A Mixed Methods Study.* Jmir Research Protocols, 2016. **5**(2).
105. Radomski, A.D., et al., *Design and delivery features that may improve the use of internet-based cognitive behavioral therapy for children and adolescents with anxiety: A realist synthesis with a persuasive systems design perspective.* Journal of Medical Internet Research, 2019. **21**(2).
106. Ragouzeos, D., et al., *"Am I OK?" using human centered design to empower rheumatoid arthritis patients through patient reported outcomes.* Patient Education and Counseling, 2019. **102**(3): p. 503-510.
107. Rai, H.K., J. Schneider, and M. Orrell, *An individual cognitive stimulation therapy app for people with dementia: Development and usability study of thinkability.* JMIR Aging, 2020. **3**(2).
108. Rai, H.K., et al., *Field-testing an iCST touch-screen application with people with dementia and carers: a mixed method study.* Aging and Mental Health, 2020.
109. Reblin, M., et al., *Development of the electronic social network assessment program using the Center for eHealth and Wellbeing Research Roadmap.* JMIR human …, 2017.
110. von Rosen, A.J., et al., *Sexual Health and the Internet: Cross-Sectional Study of Online Preferences Among Adolescents.* Journal of Medical Internet Research, 2017. **19**(11).

111. Rothgangel, A., et al., *Design and Development of a Telerehabilitation Platform for Patients With Phantom Limb Pain: A User-Centered Approach.* JMIR Rehabil Assist Technol, 2017. **4**(1): p. e2.
112. Schmidt, T., et al., *Development of an internet-delivered program and platform for the treatment of depression and anxiety in patients with ischemic heart disease in eMindYourHeart.* Informatics for Health and Social Care, 2021.
113. Scholten, M.R., S.M. Kelders, and J.E.W.C. Van Gemert-Pijnen, *An empirical study of a pedagogical agent as an adjunct to an eHealth self-management intervention: What modalities does it need to successfully support and motivate users?* Frontiers in Psychology, 2019. **10**(MAY).
114. Scholten, M.R., S.M. Kelders, and J.E.W.C. Van Gemert-Pijnen, *Self-guided Web-based interventions: Scoping review on user needs and the potential of embodied conversational agents to address them.* Journal of Medical Internet Research, 2017. **19**(11).
115. Scholten, M.R., S.M. Kelders, and J.E.W.C. van Gemert-Pijnen, *A scoped review of the potential for supportive virtual coaches as adjuncts to self-guided web-based interventions.* 2017. **10171 LNCS**: p. 43-54.
116. Sewitch, M.J., et al., *What Patients Want in a Smartphone App That Supports Colonoscopy Preparation: Qualitative Study to Inform a User-Centered Smartphone App.* Jmir Mhealth and Uhealth, 2019. **7**(7).
117. Sieverink, F., et al., *Opening the Black Box of Electronic Health: Collecting, Analyzing, and Interpreting Log Data.* 2017. **6**(8): p. e156.
118. Sjölinder, M., et al., *A multi-disciplinary approach in the development of a stroke rehabilitation tool.* … Conference on Human …, 2014.
119. Skjoth, M.M., et al., *Informed Choice for Participation in Down Syndrome Screening: Development and Content of a Web-Based Decision Aid.* Jmir Research Protocols, 2015. **4**(3).
120. Solem, I.K.L., et al., *A User-Centered Approach to an Evidence-Based Electronic Health Pain Management Intervention for People With Chronic Pain: Design and Development of EPIO.* Journal of Medical Internet Research, 2020. **22**(1).
121. Span, M., M. Hettinga, and C. Smits, *Developing a Supportive Tool to Facilitate Shared Decision Making in Dementia.* Frontiers in Aging …, 2015.
122. Span, M., C. Smits, and L.M.G.-V.D. Ven, *An interactive web tool to facilitate shared decision making in dementia: design issues perceived by caregivers and patients.* Int. J. Adv. Life …, 2014.
123. Span, M., et al., *An interactive web tool to facilitate shared decision making in dementia: Design issues perceived by caregivers and patients.* International Journal on Advances in Life Sciences, 2014. **6**(3-4): p. 107-121.
124. Span, M., C. Smits, and L.M.G.-V.D. Ven, *Towards an interactive web tool that supports shared decision making in dementia: identifying user requirements.* Int. J. Adv …, 2014.
125. Spanakis, E.G., et al., *Technology-Based Innovations to Foster Personalized Healthy Lifestyles and Well-Being: A Targeted Review.* Journal of Medical Internet Research, 2016. **18**(6).
126. Sporrel, K., et al., *The Design and Development of a Personalized Leisure Time Physical Activity Application Based on Behavior Change Theories, End-User Perceptions, and Principles From Empirical Data Mining.* Frontiers in Public Health, 2021. **8**.
127. Stara, V., et al., *Digital Health Coaching Programs Among Older Employees in Transition to Retirement: Systematic Literature Review.* Journal of Medical Internet Research, 2020. **22**(9).
128. Stuij, S.M., et al., *Developing a digital training tool to support oncologists in the skill of information-provision: a user centred approach.* Bmc Medical Education, 2020. **20**(1).

129. Stuij, S.M., et al., *Developing a digital communication training tool on information-provision in oncology: uncovering learning needs and training preferences.* BMC Med Educ, 2018. **18**(1): p. 220.
130. Suffoletto, B., et al., *Acceptability of an Opioid Relapse Prevention Text-message Intervention for Emergency Department Patients.* Journal of Addiction Medicine, 2017. **11**(6): p. 475-482.
131. Teles, S., et al., *A Multi-perspective View on AAL Stakeholders' Needs A User-centred Requirement Analysis for the Activeadvice European Project*. Proceedings of the 3rd International Conference on Information and Communication Technologies for Ageing Well and E-Health, ed. C. Rocker, et al. 2017. 104-116.
132. Tieman, J.J., et al., *Designing clinically valuable telehealth resources: processes to develop a community-based palliative care prototype.* JMIR Res Protoc, 2014. **3**(3): p. e41.
133. Timmer, M.A., et al., *A Blended Physiotherapy Intervention for Persons With Hemophilic Arthropathy: Development Study.* Journal of Medical Internet Research, 2020. **22**(6).
134. Timmerman, J.G., et al., *Co-creation of an ICT-supported cancer rehabilitation application for resected lung cancer survivors: design and evaluation.* Bmc Health Services Research, 2016. **16**.
135. van Uden-Kraan, C.F., et al., *Health-related and cancer-related Internet use by patients treated with total laryngectomy.* Supportive Care in Cancer, 2020. **28**(1): p. 131-140.
136. van der Vaart, R., et al., *Blending online therapy into regular face-to-face therapy for depression: Content, ratio and preconditions according to patients and therapists using a Delphi study.* BMC Psychiatry, 2014. **14**(1).
137. Van Velsen, L., et al., *Developing requirements for a mobile app to support citizens in dealing with ticks and tick bites via end-user profiling.* Health Informatics Journal, 2015. **21**(1): p. 24-35.
138. Van Velsen, L., et al., *Public knowledge and preventive behavior during a large-scale Salmonella outbreak: Results from an online survey in the Netherlands.* BMC Public Health, 2014. **14**(1).
139. Van Velsen, L., et al., *Should health organizations use web 2.0 media in times of an infectious disease crisis? An in-depth qualitative study of citizens' information behavior during an EHEC outbreak.* Journal of Medical Internet Research, 2012. **14**(6).
140. Verhoeven, F., et al., *Asynchronous and synchronous teleconsultation for diabetes care: A systematic literature review.* Journal of Diabetes Science and Technology, 2010. **4**(3): p. 666-684.
141. Verhoeven, F. and J. Van Gemert-Pijnen, *Discount user-centered e-health design: A quick-but-not-dirty method.* 2010. **6389 LNCS**: p. 101-123.
142. Verhoeven, F., et al., *From expert-driven to user-oriented communication of infection control guidelines.* International Journal of Human Computer Studies, 2010. **68**(6): p. 328-343.
143. Verhoeven, F., et al., *How nurses seek and evaluate clinical guidelines on the Internet.* Journal of advanced nursing, 2010. **66**(1): p. 114-127.
144. Verhoeven, F., L. Van Gemert-Pijnen, and R. Hendrix, *The Development of a web-based tool for compliance with safe work practices; A case study about methicillin-resistant staphylococcus aureus.* 2009: p. 123-128.
145. Verhoeven, F., et al., *The development of a web-based learning tool for prevention and control of MRSA.* Hygiene + Medizin, 2008. **33**(10): p. 412-416.

146. Verhoeven, F., et al., *The contribution of teleconsultation and videoconferencing to diabetes care: A systematic literature review.* Journal of Medical Internet Research, 2007. **9**(5).
147. Vermeulen, J., et al., *User-centered development and testing of a monitoring system that provides feedback regarding physical functioning to elderly people.* Patient Preference and Adherence, 2013. **7**: p. 843-854.
148. Wachtler, C., et al., *Development of a mobile clinical prediction tool to estimate future depression severity and guide treatment in primary care: User-centered design.* JMIR mHealth and uHealth, 2018. **6**(4).
149. Wahle, F., et al., *Toward the Design of Evidence-Based Mental Health Information Systems for People With Depression: A Systematic Literature Review and Meta-Analysis.* J Med Internet Res, 2017. **19**(5): p. e191.
150. Walters, L.E.M., R.E. Scott, and M. Mars, *Design requirements for a teledermatology scale-up framework.* South African Computer Journal, 2018.
151. Walters, L.E., M. Mars, and R.E. Scott, *A Review and Critique of Teledermatology in the South African Public Health Sector.* Stud Health Technol Inform, 2016. **231**: p. 143-151.
152. Wentink, M., L.v. Bodegom-Vos, and B. Brouns, *How to improve eRehabilitation programs in stroke care? A focus group study to identify requirements of end-users.* BMC medical informatics …, 2019.
153. Wentink, M.M. and L.v. Bodegom-Vos, *What is important in E-health interventions for stroke rehabilitation? A survey study among patients, informal caregivers and health professionals.* International journal …, 2018.
154. Wentzel, J., et al., *Card sorting to evaluate the robustness of the information architecture of a protocol website.* International Journal of Medical Informatics, 2016. **86**: p. 71-81.
155. Wentzel, J., et al., *Participatory eHealth development to support nurses in antimicrobial stewardship.* BMC Medical Informatics and Decision Making, 2014. **14**(1).
156. Wentzel, M.J., et al., *Towards an Internet-based Infectious Disease Management Platform to Increase Patient Safety*. 2011. 47-50.
157. Whiteside, U., et al., *Designing messaging to engage patients in an online suicide prevention intervention: Survey results from patients with current suicidal ideation.* Journal of Medical Internet Research, 2014. **16**(2).
158. Winterling, J., et al., *Development of a self-help web-based intervention targeting young cancer patients with sexual problems and fertility distress in collaboration with patient research partners.* JMIR research protocols, 2016. **5**(2): p. e60.
159. van Woezik, A.F.G., et al., *Tackling wicked problems in infection prevention and control: A guideline for co-creation with stakeholders.* Antimicrobial Resistance and Infection Control, 2016. **5**(1).
160. Yap, M.B., et al., *Partners in Parenting: A Multi-Level Web-Based Approach to Support Parents in Prevention and Early Intervention for Adolescent Depression and Anxiety.* JMIR Ment Health, 2017. **4**(4): p. e59.
